# Checklist for the crop weeds of Paraguay

**DOI:** 10.3897/phytokeys.73.10135

**Published:** 2016-10-18

**Authors:** Juana De Egea, Fátima Mereles, María del Carmen Peña-Chocarro, Gloria Céspedes

**Affiliations:** 1 Associate Researcher for the Centro para el Desarrollo de la Investigación Científica. Fundación Moisés Bertoni para la Conservación de la Naturaleza y Laboratorios Díaz-Gill, Manduvirá 635, Asunción, Paraguay; 2 Researcher for the Programa Nacional de Incentivo a Investigadores del Consejo Nacional de Ciencia y Tecnología (CONACYT), Paraguay; 3 The Natural History Museum, Cromwell Rd, London SW75BD, U.K.; 4 Asociación Etnobotánica Paraguaya, Julia Miranda Cueto de Estigarribia 795, Edificio Mariucci, 2° piso, oficina 208, San Lorenzo, Paraguay

**Keywords:** Weeds, crops, Paraguay, agro-ecosystems, new records

## Abstract

Paraguay, a country whose economy is based mainly on agriculture and livestock for export, has experienced a major expansion in mechanized crops during the last few decades. Despite being heavily dependent on agriculture, Paraguay has very limited research on crop weeds, in spite of these having a high economic impact on production. This work aims to update and enhance the knowledgebase on the most common weeds affecting productive fields throughout the different ecoregions of Paraguay. We present here the first checklist of crop weeds for the country, which includes a total of 256 taxa (189 species, 10 subspecies, 54 varieties and 3 forms), with the most species-rich families being Poaceae and Asteraceae followed by Malvaceae, Amaranthaceae, Fabaceae and Solanaceae. The list includes three new records for the country. Synonyms, distribution details within Paraguay, habit and a voucher specimen are provided for each taxon.

## Introduction

Weeds can be defined as plants (not necessarily alien) that grow on sites where they are not wanted and which have a detectable economic and/or environmental impact (Richardson et al. 2000, Pyšek et al. 2004). They overlap partly with invasive plants, which are introduced species that cause problems, either in agriculture or in natural areas. According to Pyšek et al. (2004), there have been terminological misunderstandings in studies dealing with plant invasions, partly caused by different perceptions of plant invasions by particular biological disciplines and viewpoints. As shown by these authors, plants encroaching on habitats in which they were not present before can be assessed from the ecological point of view (and termed *colonizers*), from the biogeographical point of view (*invaders*, or *alien plants* in a more general sense), or from the anthropocentric point of view (termed *weeds*, harmful species, problem plants, noxious plants, pests, etc.).

Paraguay has a total area of 406,752 km^2^ and is divided into two regions by the Río Paraguay: the Oriental Region and the Occidental Region. The economy is based mainly on agriculture and livestock for export; between the years 2013–2014, approximately 52,381 km^2^ were dedicated to temporary and permanent crops (DGEEC 2015), particularly soybean (35,000 km^2^), corn (8,000 km^2^) and wheat (5,600 km^2^). During the second half of the twentieth century, global soy production grew tenfold from 27 million tons to 269 million tons (WWF 2016). This increased production was the result of a major expansion in mechanized crops, including other crops that form part of this complex such as wheat, corn, sorghum, canola, among others. It is expected that production will double by 2050 (Bruinsma 2009, WWF 2014). Indeed, Paraguay plays a strong role in this soybean industry; during the 2012–2013 harvest, Paraguay ranked sixth as a global producer and fourth in exports. Itapúa and Alto Paraná have been the highest producing departments, while expansion currently occurs in the departments of San Pedro, Canindeyú and Alto Paraguay (WWF 2016).

Agriculture in Paraguay is carried out on a variety of very distinct soil types in both natural regions. The soils in the Oriental Region are for the most part reddish in colour, resulting from geological events during the Precambrian era and Jurassic and Cretaceous periods (Bertoni and Gorham 1973). These soils include the leptosols and arenosols, which are generally poor in nutrients, and the loamy sand and sandy loam regosols, which are rich in nutrients and where most of the mechanized crops in the region have been carried out (González Erico 2007). In the Occidental Region, most of the soil types are brackish, with a variable content of clay, including planosols, solonetz, gleysols and regosols, where mechanized crops are carried out (Proyecto Sistema Ambiental del Chaco 1992–1997).

Despite being mainly an agricultural country, Paraguay does not have much in terms of research on crop weeds, even in cases where these have a high economic impact on production. Only two sources can actually be considered as references on the subject: Lurvey (1983), the only weed guide available for the country, and the *Flora del Paraguay* collection (Spichiger and Bocquet 1983–1985, Spichiger 1987–1989, Spichiger and Ramella 1990–2002, Ramella and Perret 2008–2014) that superficially covers the behavior of some species, in terms of their weediness, within the families included in the collection. Even herbarium data has proved to be of little use to determine the level of weediness of a species, due to the well-known biases in plant collection towards natural areas.

Weeds have a particular set of skills for survival through mechanisms such as high competitive ability, high seed production, seed production for as long as growing conditions permit, rapid growth throughout vegetative phase to flowering, germination requirements fulfilled in many environments, adaptations for short and long dispersal and great longevity of seeds, self-compatible but not completely autogamous or apomictic, and when cross-pollinated, either pollinated by unspecialized visitors or by wind (Baker 1965, Baker 1974, Lorenzi 2000). Their development in productive areas tends to reduce the quality and quantity of the crop yield, making it difficult to harvest, and in some extreme cases even making it unviable (Guglieri-Caporal et al. 2011). According to Pott et al. (2006), success in controlling invasive plants begins with the floristic inventory of the infesting species and knowledge of the biology of those species that are predominant.

Because of their numerous interactions with humans, their biology, ecology, evolution and community dynamics, weeds are an important subject of study (Kuester et al. 2014). In fact, the study of plant species considered to be weeds in agro-ecosystems is a subject of high interest in the area of invasion ecology, not only because of their natural ability to become established in natural and semi-natural environments (Figueroa et al. 2004, Pyšek et al. 2004) with the consequent great ecological harm they can cause to ecosystems, but also because of the special attributes that make these plant species more invasive (Rejmánek and Richardson 1996), and the way they respond to human disturbance (Hill et al. 2002). Other studies have also focused on revealing taxonomic patterns among natural area invaders and agricultural weeds (Daehler 1998, Kuester et al. 2014), while Conservation Agriculture builds upon weed ecology to generate integrated and sustainable weed management strategies (Bajwa 2014).

Regarding the increasing importance of weed science in a country such as Paraguay, which depends heavily on agricultural production, this work aims to update and enhance the knowledgebase of the most common weeds affecting productive fields throughout the different ecoregions of the country, as well as the generation of relevant information to facilitate their identification and management.

## Methods

Paraguay is a land-locked country located between 19° and 28° south latitude and 54° and 63° west longitude at the heart of the South American continent, and lies entirely within the Río de la Plata drainage system, second only in size to the Amazon basin. The country is divided into two regions by the Río Paraguay: the Oriental Region, or eastern region, also known as the Paraná region, and the Occidental Region, or western region, also known as the Chaco (part of the Gran Chaco Americano shared by Argentina, Bolivia and Paraguay). Paraguay is divided into 17 departments, 14 of which are located to the east of the Río Paraguay (De Egea et al. 2012).

**Figure 1.**
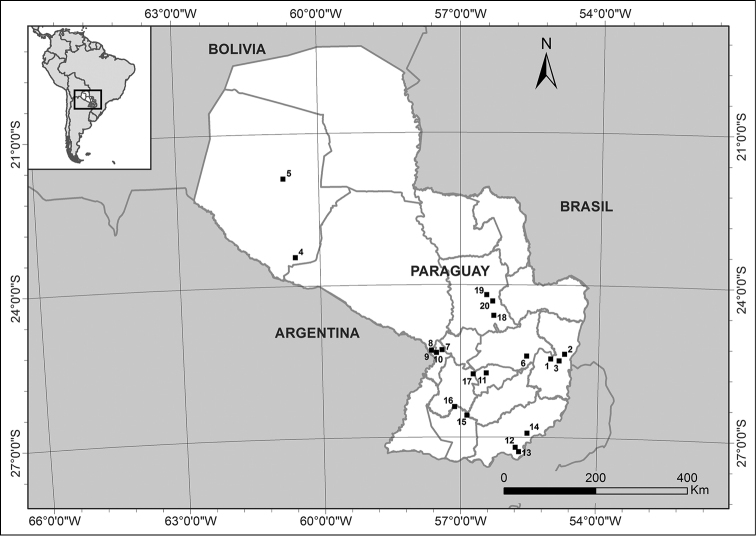
Location of the crop weed collection sites

While building the checklist, we included: 1) a survey of all plant species occurring in key sites, mainly in areas of mechanized soybean, sorghum, sugarcane, corn and rice, where weed management and control was being periodically implemented (Figure 1, Table 1). Each site was surveyed once, at a time when crop fields presented a high level of weed infestation and prior to any weed control activities. Sampling was carried out between 1–3 days, depending on the size of the available crop area per site, by surveying a series of random points (up to 8 per day) to collect the weeds occurring within a 50 meter radius. The data collection included the random collection of plant specimens, georeferencing of the records, the identification of specimens and the resolution of taxonomic problems. Where possible, specimens were collected in quadruplicate; the set of originals were stored in FCQ and duplicates were sent to BM, G and CTES; and 2) the systematization of data available in the literature. Three sources were considered as reference: Lurvey (1983), the *Flora del Paraguay* collection, and the database of The International Survey of Herbicide Resistant Weeds (Heap 2016), containing a list of the more resistant weed species in the region. Online databases and herbarium resources such as TROPICOS (2016) and the *Catálogo de las plantas vasculares del Cono Sur* (Zuloaga and Belgrano 2015) were also consulted.

**Table 1.** Crop weed collection sites.

Map key	Department	Locality	Type of habitat/crop
**1**	**Alto Paraná**	Colonia Yguazú, Centro de Investigación Agropecuaria del Paraguay	Soybean crops
**2**	**Alto Paraná**	Hernandarias	Soybean crops
**3**	**Alto Paraná**	Minga Guazú, Universidad Nacional del Este (UNE) experimental plots	Soybean crops
**4**	**Boquerón**	Estancia Toro Mocho	Cattle pastures and forest edges
**5**	**Boquerón**	Mariscal Estigarribia, Estancia Jeroviá	Soybean and sorghum crops
**6**	**Caaguazú**	Juan Eulogio Estigarribia	Soybean crops
**7**	**Central**	Areguá	Strawberry crops
**8**	**Central**	Asunción	Vegetable patch/home garden
**9**	**Central**	Asunción	Disturbed soils
**10**	**Central**	San Lorenzo, Universidad Nacional de Asunción, experimental plot	Potato, tomato and cabbage crops
**11**	**Guairá**	Villarrica	Disturbed soils
**12**	**Itapúa**	Capitán Miranda	Soybean crops
**13**	**Itapúa**	Nueva Alborada District	Soybean crops
**14**	**Itapúa**	Pirapó	Soybean crops
**15**	**Misiones**	San Miguel, Establecimiento La Graciela	Rice crops
**16**	**Misiones**	Villa Florida	Disturbed soils
**17**	**Paraguarí**	Villarrica – Paraguarí road	Sugarcane crops
**18**	**San Pedro**	Barrio San Pedro, Dekalpar crops	Corn crops
**19**	**San Pedro**	Cruce Liberación	Disturbed soils
**20**	**San Pedro**	Instituto Paraguayo de Tecnología Agrícola (IPTA) experimental plots	Fruit crops

Data was compiled in a MS Access database. Flowering plant family circumscription follows APG III (Stevens 2001 onwards) and pteridophyte classification follows Smith et al. (2006). In the checklist, families are sorted alphabetically and genera are sorted alphabetically within families. For each taxon we present the accepted name, place and date of publication, synonyms, habit, general distribution in Paraguay, residence status (all species are native except where indicated), and one voucher specimen. Relevant bibliographic citacions are included for taxa recorded in literature, but not found during field surveys nor in the consulted herbaria (BM, CTES, FCQ, and PY). Synonymy follows Zuloaga and Belgrano (2015), except for those taxa covered in more recent literature. Herbarium acronyms used in the text follow Index Herbariorum (Thiers, continuously updated). New reports of taxa for Paraguay are marked with an arrowhead (►) and those taxa not cited in Zuloaga and Belgrano (2015) for Paraguay but recorded in other publications or databases are marked with an asterisk (*).

## Data resources

All occurrence data underpinning the checklist have been uploaded to the Natural History Museum Data Portal (https://doi.org/10.5519/0060042) and are provided as a data supplement to this paper.

## Results and discussion

The list includes a total of 256 taxa (189 species, 10 subspecies, 54 varieties and 3 formas), making up 38 families and 141 genera, of which all except one are angiosperms. There is only one pteridophyte listed, *Pteridium arachnoideum* (Kaulf.) Maxon, a very close relative of *Pteridium aquilinum* (L.) Kuhn, a cosmopolitan species with a vigorous vegetative reproductive system (Baker 1974), which is a very common weed worldwide.

The most species-rich families are Poaceae, Asteraceae and Malvaceae, followed by Amaranthaceae, Fabaceae, Solanaceae, Polygonaceae and Cyperaceae (see Table 2). According to Baker (1974), Malvaceae, Amaranthaceae, Cyperaceae and Poaceae are the families which have a higher number of weed species in warm regions, in line with our results.

**Figure 2.**
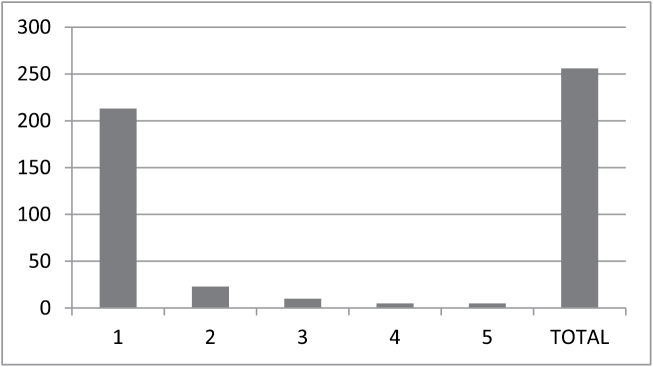
Life forms of the taxa included. **1** herbs **2** subshrubs **3** shrubs **4** small trees **5** vines.

Over eighty-three percent (213) of the species in the Checklist are herbs, 14.8% (38) woody species (small trees, shrubs or subshrubs) and only 1.95% (5) are vines (Figure 2).

**Table 2.** Number of genera and taxa in the largest families.

Families	Genera	Taxa
Poaceae	22	50
Asteraceae	29	41
Malvaceae	11	20
Amaranthaceae	4	13
Fabaceae	9	12
Solanaceae	3	12
Polygonaceae	2	11
Cyperaceae	4	10
Euphorbiaceae	5	10
Other families	52	77

The majority of the listed taxa are native to Paraguay (209), representing 81.64% of the total, including one endemic species (*Turnera grandidentata* (Urb.) Arbo), while the remaining 47 taxa are introduced. Families with the highest number of non-native species are Poaceae with 16 taxa followed by Brassicaceae with 4 taxa. Most introduced species remain within the status of established plants, except for some species which also show invasive behaviour. Such is the case of *Calotropis procera* (Aiton) W.T.Aiton, reported for the first time in Paraguay in 1994 (Mereles and Degen 1994), found in cattle pastures in northern Paraguay, and currently quite frequent as a weed of difficult eradication within the dry Chaco. *Urochloa brizantha* (Hochst. ex A.Rich.) R.D.Webster and *Urochloa decumbens* (Stapf) R.D.Webster are also two species that were introduced for grazing (Zuloaga et al. 2014), and are now found in both natural and agro-ecosystems. These three species were also identified as invasive species in Brazil (Base de dados nacional de espécies exóticas invasoras I3N Brasil 2016).

The list includes three new records for the country, collected during crop surveys (Avena sativa (J.Presl.) Barkworth subsp. sativa, Cyperus eragrostis Lam. var. eragrostis and *Leonurus cardiaca* L. It also includes another seven taxa not listed in Zuloaga and Belgrano (2015), as being present in Paraguay, but recorded in TROPICOS (2016) or in recent publications (De Egea et al. 2016): Amaranthus hybridus L. subsp. cruentus (L.) Thell., Avena sativa L. subsp. sativa, *Capsella bursa-pastoris* (L.) Medik., *Commelina benghalensis* L., Cyperus eragrostis Lam. var. eragrostis, *Glandularia cabrerae* (Moldenke) Botta, *Leonurus cardiaca* L., *Oldenlandia lancifolia* (Schumach.) DC., *Tragus australianus* and *Tridax procumbens* L.

Eight taxa mentioned in literature, but not found during field surveys nor in the consulted herbaria, were also included in the checklist: *Capsella bursa-pastoris* (L.) Medik., *Lepidium virginicum* L., *Raphanus raphanistrum* L. (Lurvey 1983), Digitaria aequiglumis (Hack. & Arechav.) Parodi var. aequiglumis, *Echinochloa oryzoides* (Ard.) Fritsch (Zuloaga et al. 1994), *Paspalum urvillei* Steud., *Setaria scandens* Schrad. (Zuloaga et al. 2014), and *Polygonum aviculare* L. (Cialdella and Brandbyge 2001).

Nine species were found to be the most frequent and abundant within all crops surveyed: *Amaranthus hybridus* L., *Bidens pilosa* L., *Conyza bonariensis* (L.) Cronquist, *Commelina erecta* L., *Eleusine indica* (L.) Gaertn., *Euphorbia heterophylla* L., *Digitaria insularis* (L.) Fedde, *Richardia brasiliensis* Gomes, and *Solanum americanum* Mill. These species were found in both natural regions of Paraguay and on different soil types, showing a greater plasticity/resilience than the other species found. Overall, they are species with high adaptability, fast growth, resistance to drought and high biomass and seed production. It is also likely that the seeds of these weed species accompany the seeds of the harvested grain crops. All these have been listed and highlighted in regional literature on weeds as being problematic species (Ferrari 2012, Guglieri-Caporal 2011, Lorenzi 2000), and with the exception of *Commelina erecta* and *Richardia brasiliensis*, are considered among the weeds with greatest herbicide resistance according to The International Survey of Herbicide Resistant Weeds (Heap 2016).

## Checklist

### FERNS

#### DENNSTAEDTIACEAE

***Pteridium arachnoideum*** (Kaulf.) Maxon, J. Wash. Acad. Sci. 14: 89. 1924.

Syn.: Pteridium aquilinum (L.) Kuhn var. arachnoideum (Kaulf.) Brade, Pteridium aquilinum ssp. psittacinum (C.Presl) C.Chr, Pteridium aquilinum var. umbrosum H.Christ, nom. illeg., Pteridium caudatum (L.) Maxon ssp. arachnoideum (Kaulf.) Lellinger, Pteridium esculentum (G.Forst.) Cockayne ssp. arachnoideum (Kaulf.) J.A.Thomson, *Pteridium psittacinum* (C.Presl) Maxon, Pteris aquilina L. var. arachnoidea (Kaulf.) D.C.Eaton, Pteris aquilina var. psittacina (C.Presl) Baker, *Pteris arachnoidea* Kaulf., *Pteris esculenta* G.Forst., *Pteris psittacina* C.Presl.

Herb.

Departmental distribution: Amambay, Caazapá, Canindeyú, Cordillera, Guairá, Paraguarí, San Pedro.

Voucher: *J. De Egea et al. 1340* (BM, FCQ, G).

### SEED PLANTS

#### ACANTHACEAE

***Justicia squarrosa*** Griseb., Abh. Königl. Ges. Wiss. Göttingen 19: 226. 1874.

Syn.: *Beloperone ciliata* (Nees) Hook.f., *Beloperone squarrosa* (Griseb.) Lindau, *Dianthera ciliata* (Nees) Benth. & Hook.f., hom. illeg., *Jacobinia ciliata* Nees.

Herb or subshrub.

Departmental distribution: Alto Paraguay, Boquerón, Presidente Hayes.

Voucher: *F. Mereles & S. Soria 9978* (FCQ).

#### ALISMATACEAE

***Sagittaria montevidensis*** Cham. & Schltdl. subsp. ***montevidensis***, Linnaea 2: 156. 1827.

Syn.: *Sagittaria andina* Phil., Sagittaria montevidensis f. normalis Hauman, nom. inval., *Sagittaria multinervia* Larrañaga, Sagittaria pugioniformis L. var. montevidensis (Cham. & Schltdl.) Kuntze

Herb.

Departmental distribution: Alto Paraguay, Amambay, Boquerón, Caaguazú, Caazapá, Central, Concepción, Cordillera, Guairá, Itapúa, Misiones, Ñeembucú, Paraguarí, Presidente Hayes, San Pedro.

Voucher: *G. Céspedes et al. 445* (BM, FCQ).

#### AMARANTHACEAE

***Alternanthera ficoidea*** (L.) P.Beauv., Fl. Oware 2: 66. 1818.

Syn.: Alternanthera polygonoides (L.) R.Br. ex Griseb. var. diffusa (Mart.) Hicken, *Alternanthera tenella* Colla, *Bucholzia ficoidea* (L.) Mart., *Gomphrena ficoidea* L.

Herb.

Departmental distribution: Alto Paraguay, Alto Paraná, Amambay, Central, Presidente Hayes, San Pedro.

Voucher: *J. De Egea et al. 1230* (FCQ).

***Alternanthera paronichyoides*** A.St.-Hil. subsp. ***chacoënsis*** (Morong ex Morong & Britton) Pedersen, Darwiniana 14: 440. 1967.

Syn.: *Alternanthera chacoënsis* Morong ex Morong & Britton, Alternanthera ficoidea (L.) Sm. ssp. chacoënsis (Morong ex Morong & Britton) Pedersen, *Alternanthera morongii* Uline, Alternanthera paronychioides var. chacöensis (Morong ex Morong & Britton) Pedersen, Alternanthera paronychioides var. robusta Chodat.

Herb.

Departmental distribution: Alto Paraguay, Boquerón, Caaguazú, Central, Concepción, Cordillera, Guairá, Ñeembucú, Paraguarí, Presidente Hayes.

Voucher: *F. Mereles et al. 10060* (FCQ).

***Alternanthera philoxeroides*** (Mart.) Griseb. f. ***philoxeroides***, Abh. Königl. Ges. Wiss. Göttingen 24: 36. 1879.

Syn.: *Achyranthes paludosa* Bunbury, *Achyranthes philoxeroides* (Mart.) Standl., Alternanthera philoxeroides (Mart.) Griseb. var. acutifolia (Mart.) Hicken, Alternanthera philoxeroides var. obtusifolia (Moq.) Hicken, *Bucholzia philoxeroides* Mart., Bucholzia philoxeroides var. obtusifolia Mart., *Mogiphanes philorexoides* D.Parodi, *Telanthera philoxeroides* (Mart.) Moq., Telanthera philoxeroides var. denticulata Seub., Telanthera philoxeroides var. obtusifolia Moq.

Herb aquatic.

Departmental distribution: Alto Paraguay, Central, Presidente Hayes.

Voucher: *N. Soria 332* (FCQ).

*Amaranthus hybridus L. subsp. ***cruentus*** (L.) Thell., Fl. Adv. Montpellier 205. 1912.

Syn.: *Amaranthus cruentus* L., *Amaranthus flavus* L., *Amaranthus paniculatus* L.

Herb. Introduced.

Departmental distribution: Alto Paraguay, Guairá, San Pedro.

Voucher: *F. Mereles & S. Soria 10212* (FCQ).

***Amaranthus hybridus*** L. subsp. ***hybridus***, Sp. Pl. 2: 990. 1753.

Syn.: *Amaranthus chlorostachys* Willd., Amaranthus hybridus L. var. hypochondriacus (L.) Rob., Amaranthus hybridus var. quitensis (Kunth) Covas, Amaranthus hypochondriacus L. var. hypochondriacus, *Amaranthus quitensis* Kunth, Amaranthus quitensis var. stuckertianus Thell.

Herb. Introduced.

Departmental distribution: Alto Paraguay, Boquerón, Central, Guairá, Itapúa, Ñeembucú, Presidente Hayes, San Pedro.

Voucher: *F. Mereles & S. Soria 9946* (FCQ).

***Amaranthus muricatus*** (Moq.) Hieron., Pl. Diaph. Fl. Argent. 227. 1882.

Syn.: *Euxolus muricatus* Moq.

Herb.

Departmental distribution: Alto Paraguay, Boquerón, Presidente Hayes, San Pedro.

Voucher: *J. De Egea et al. 1243* (BM, FCQ).

***Amaranthus standleyanus*** Parodi ex Covas, Darwiniana 5: 339. 1941.

Syn.: *Amaranthus parodii* Standl., hom. illeg., *Amaranthus vulgatissimus* sensu Thell. non Speg.

Herb.

Departmental distribution: Alto Paraguay, Boquerón, Misiones, Presidente Hayes.

Voucher: *F. Mereles et al. 10174* (BM, CTES, FCQ, G).

***Amaranthus viridis*** L., Sp. Pl., ed. 2. 2: 1405. 1763.

Syn.: *Amaranthus gracilis* Desf., *Chenopodium caudatum* Jacq., *Euxolus caudatus* (Jacq.) Moq., *Euxolus viridis* (L.) Moq.

Herb.

Departmental distribution: Alto Paraguay, Boquerón, Central, Concepción, Guairá, Paraguarí, Presidente Hayes, San Pedro.

Voucher: *F. Mereles & S. Soria 10186* (FCQ).

***Dysphania ambrosioides*** (L.) Mosyakin & Clemants, Ukrayins’k. Bot. Zhurn. 59(4): 382. 2002.

Syn.: *Ambrina ambrosioides* (L.) Spach, *Ambrina anthelmintica* Spach, *Ambrina parvula* Phil., *Chenopodium ambrosioides* L., Chenopodium ambrosioides var. dentata Fenzl, Chenopodium ambrosioides f. dentatum (Fenzl) Aellen, Chenopodium ambrosioides ssp. euambrosioides Aellen, Chenopodium ambrosioides var. genuinum Willk., Chenopodium ambrosioides f. genuinum (Willk.) Aellen, Chenopodium ambrosioides var. integrifolium Fenzl, Chenopodium ambrosioides f. integrifolium (Fenzl) Aellen, Chenopodium ambrosioides var. pinnatifidum Willk., Chenopodium ambrosioides f. pinnatifidum (Willk.) Aellen, Chenopodium ambrosioides f. rotundantum Aellen, Chenopodium ambrosioides var. suffruticosum (Willd.) Asch. & Graebn., Chenopodium ambrosioides var. typica Speg., Chenopodium ambrosioides var. typicum (Speg.) Aellen, *Chenopodium suffruticosum* Willd., Chenopodium suffruticosum ssp. remotum Vorosh., *Teloxys ambrosioides* (L.) W.A.Weber, *Ambrina incisa* Phil.

Herb.

Departmental distribution: Alto Paraguay, Amambay, Caaguazú, Caazapá, Central, Concepción, Cordillera, Guairá, Itapúa, Paraguarí, Presidente Hayes.

Voucher: *R. Degen 120* (FCQ).

***Gomphrena celosioides*** Mart. f. ***aureiflora*** (Chodat) Pedersen, Darwiniana 20: 272. 1976.

Syn.: Gomphrena celosioides var. aureiflora Stuchlik, Gomphrena celosioides f. grandifolia Stuchlik, Gomphrena celosioides f. parvifolia Stuchlik, Gomphrena decumbens Jacq. f. aureiflora Chodat, Gomphrena decumbens var. aureiflora (Chodat) Stuchlik, Gomphrena hygrophila Mart. f. luteiflora Herzog.

Herb.

Departmental distribution: Guairá, Ñeembucú, Paraguarí, Presidente Hayes.

Voucher: *J. De Egea et al. 1316* (BM, FCQ).

***Gomphrena celosioides*** Mart. var. ***celosioides***, Nova Acta Phys.-Med. Acad. Caes. Leop.-Carol. Nat. Cur. 13: 301. 1826.

Syn.: Gomphrena decumbens Jacq. f. albiflora Chodat & Hassl., Gomphrena decumbens var. albiflora (Chodat & Hassl.) Stuchlik, Gomphrena decumbens var. genuina Stuchlik, nom. inval., Gomphrena perennis L. f. ramosissima Stuchlik, *Xeraea celosioides* (Mart.) Kuntze

Herb.

Departmental distribution: Alto Paraguay, Alto Paraná, Boquerón, Caaguazú, Caazapá, Central, Cordillera, Guairá, Itapúa, Ñeembucú, Presidente Hayes, San Pedro.

Voucher: *F. Mereles et al. 9913* (FCQ).

***Gomphrena elegans*** Mart. var. ***elegans***, Nov. Gen. Sp. Pl. 2(1): 17, t. 119. 1826.

Syn.: Gomphrena elegans Mart. var. genuina Stuchlik, nom. inval., Gomphrena elegans f. nigro-virida Stuchlik, *Gomphrena hilariana* Moq., *Gomphrena viridifolia* Suess., *Xeraea elegans* (Mart.) Kuntze, *Xeraea hilariana* (Moq.) Kuntze

Herb.

Departmental distribution: Canindeyú, Cordillera, Guairá, Paraguarí.

Voucher: *E. Zardini & R. Velázquez 11556* (FCQ).

***Gomphrena perennis*** L. var. ***perennis***, Sp. Pl. 1: 224. 1753.

Syn.: Gomphrena perennis L. ssp. genuina Stuchlik, nom. inval., Gomphrena perennis f. parvifolia, Gomphrena perennis f. villosa, *Gomphrena villosa* Mart., *Xeraea perennis* (L.) Kuntze, *Xeraea villosa* (Mart.) Kuntze

Herb.

Departmental distribution: Alto Paraguay, Boquerón.

Voucher: *F. Mereles & S. Soria 9940* (BM, CTES, FCQ, G).

#### APIACEAE

***Cyclospermum leptophyllum*** (Pers.) Sprague var. ***leptophyllum***, J. Bot. 61: 131. 1923.

Syn.: *Apium ammi* Urb., Apium ammi f. filamentosum (Kuntze) H.Wolff, Apium ammi var. filamentosum Kuntze, Apium ammi Urb. var. genuinum H.Wolff, Apium ammi var. leptophyllum (Pers.) Kuntze, Apium ammi f. nanum Kuntze, Apium ammi f. pedunculata Chodat.

Apium laciniatum (DC.) Urb. f. elatius (Hook. & Arn.) H.Wolff, *Apium leptophyllum* (Pers.) F.Muell. ex Benth., *Apium ranunculifolium* auct. non Kunth, *Apium ranunculifolium* (DC.) Reiche, comb. illeg., Helosciadium laciniatum DC. var. elatius Hook. & Arn., *Helosciadium leptophyllum* (Pers.) DC., *Helosciadium ranunculifolium* DC., *Pimpinella leptophylla* Pers.

Herb.

Departmental distribution: Alto Paraguay, Alto Paraná, Boquerón, Caazapá, Central, Guairá, Ñeembucú, Presidente Hayes, San Pedro.

Voucher: *J. De Egea et al. 1321* (FCQ).

#### APOCYNACEAE

***Asclepias curassavica*** L., Sp. Pl. 1: 215. 1753.

Herb. Introduced.

Departmental distribution: Amambay, Canindeyú, Central, Cordillera, Guairá, Itapúa, Misiones, Paraguarí, Presidente Hayes.

Voucher: *F. Mereles et al. 10011* (BM, FCQ, G).

***Asclepias mellodora*** A.St.-Hil., Pl. Rem. Bres. 227. 1824.

Syn.: *Asclepias campestris* Decne., Asclepias campestris var. angustifolia Kuntze, Asclepias campestris var. schlechteri Kuntze, *Asclepias curupi* E.Fourn., *Asclepias jangadensis* S.Moore, Asclepias marginata Decne. var. bodenbenderi Kuntze, Asclepias mellodora var. bodenbenderi (Kuntze) Bollwinkel, Asclepias mellodora var. minor A.St.-Hil., Asclepias mellodora var. multinervis (E.Fourn.) Bollwinkel, *Asclepias multinervis* E.Fourn., *Asclepias nervosa* Decne., *Asclepias papillosa* Silveira, *Asclepias umbellata* Vell.

Herb.

Departmental distribution: Amambay, Boquerón, Caazapá, Canindeyú, Central, Concepción, Cordillera, Guairá, Itapúa, Misiones, Ñeembucú, Paraguarí, Presidente Hayes, San Pedro.

Voucher: *J. De Egea et al. 1329* (FCQ).

***Calotropis procera*** (Aiton) W.T.Aiton, Hort. Kew., ed. 2 2: 78. 1811.

Syn.: *Asclepias procera* Aiton

Shrub or small tree. Introduced.

Departmental distribution: Alto Paraguay, Boquerón.

Voucher: *F. Mereles & S. Soria 9983* (FCQ, G).

#### ARALIACEAE

***Hydrocotyle bowlesioides*** Mathias & Constance, Bull. Torrey Bot. Club 69: 151. 1942.

Herb.

Departmental distribution: Alto Paraná, Amambay, Caazapá, Central, Guairá, Paraguarí.

Voucher: *F. Mereles et al. 10098* (FCQ).

#### ASTERACEAE

***Acanthospermum hispidum*** DC., Prodr. 5: 522. 1836.

Syn.: Acanthospermum humile DC. var. hispidum (DC.) Kuntze

Herb.

Departmental distribution: Alto Paraguay, Boquerón, Central, Concepción, Cordillera, Ñeembucú, Paraguarí, Presidente Hayes.

Voucher: *A. Quarti P050* (FCQ).

***Ageratum conyzoides*** L., Sp. Pl. 2: 839. 1753.

Syn.: Ageratum conyzoides L. var. hirtum (Lam.) DC., *Ageratum hirsutum* Poir., *Ageratum hirtum* Lam., *Ageratum latifolium* Cav., *Carelia conyzoides* (L.) Kuntze

Herb.

Departmental distribution: Amambay, Caazapá, Canindeyú, Central, Cordillera, Guairá, Itapúa, San Pedro.

Voucher: *J. De Egea et al. 1276* (FCQ).

***Ambrosia elatior*** L., Sp. Pl. 2: 987. 1753.

Syn.: *Ambrosia artemisiifolia* L., Ambrosia artemisiifolia var. elatior (L.) Descourt., *Ambrosia chilensis* Hook. & Arn., *Ambrosia peruviana* Cabrera, hom. illeg.

Herb.

Departmental distribution: Alto Paraná, Amambay, Boquerón, Caazapá, Central, Concepción, Cordillera, Guairá, Presidente Hayes, San Pedro.

Voucher: *F. Mereles et al. 10090* (FCQ, G).

***Ambrosia tenuifolia*** Spreng., Syst. Veg. (ed. 16) 3: 851. 1826.

Herb.

Departmental distribution: Central, Paraguarí, Presidente Hayes.

Voucher: *F. Mereles 5263* (CTES, FCQ).

***Aspilia montevidensis*** (Spreng.) Kuntze var. ***montevidensis***, Revis. Gen. Pl. 3[3]: 129. 1898.

Syn.: *Aspilia arillata* (DC.) Griseb., *Aspilia buphthalmiflora* (DC.) Griseb., *Aspilia calendulacea* (DC.) Griseb., Aspilia montevidensis (Spreng.) Kuntze var. setosa (Griseb.) Cabrera, *Aspilia setosa* Griseb., *Leighia arillata* DC., *Leighia buphthalmiflora* DC., *Leighia calendulacea* DC., *Verbesina montevidensis* Spreng., *Wedelia montevidensis* (Spreng.) B.L.Turner

Herb.

Departmental distribution: Amambay, Caaguazú, Caazapá, Canindeyú, Central, Concepción, Cordillera, Guairá, Itapúa, Misiones, Paraguarí, Presidente Hayes, San Pedro.

Voucher: *J. De Egea et al. 1334* (BM, CTES, FCQ, G).

***Aspilia silphioides*** Benth. & Hook. f., Gen. Pl. 2(1): 372. 1873.

Syn.: *Gymnopsis helianthoides* DC., *Leighia silphioides* Hook. & Arn., *Wedelia silphioides* (Hook. & Arn.) B.L.Turner

Herb.

Departmental distribution: Amambay, Boquerón, Central, Concepción, Cordillera, Ñeembucú, Presidente Hayes.

Voucher: *F. Mereles & S. Soria 9953* (BM, FCQ, G).

***Baccharis salicifolia*** (Ruiz & Pav.) Pers., Syn. Pl. 2: 425. 1807.

Syn.: *Baccharis alamanii* DC., *Baccharis araucana* Phil., *Baccharis pallida* Heering ex Reiche, *Baccharis chilquilla* DC., nom. superfl., *Baccharis coerulescens* DC., Baccharis confertifolia Colla var. confertifolia, *Baccharis cuervii* Phil., *Baccharis fevillei* DC., Baccharis glutinosa Pers. var. incisa Heering, *Baccharis huydobriana* J.Rémy, *Baccharis iresinoides* Kunth, *Baccharis kraussei* Heering ex Reiche, *Baccharis lanceolata* Kunth emend. Heering, *Baccharis linifolia* DC., hom. illeg., *Baccharis longifolia* DC., *Baccharis longipes* Kunze ex DC., *Baccharis marginalis* DC., Baccharis marginalis var. araucana (Phil.) Heering, Baccharis marginalis var. linifolia (Phil.) Heering, Baccharis marginalis var. longipes (Kunze ex DC.) Heering, *Baccharis parviflora* (Ruiz & Pav.) Pers., *Baccharis purpurascens* Heering, *Pingraea salicifolia* (Ruiz & Pav.) F.H.Hellwig, Baccharis salicifolia (Ruiz & Pav.) Pers. var. longifolia (DC.) Cuatrec., Baccharis sphaerocephala Hook. & Arn. var. krausei (Heering) Malag., comb. illeg., Baccharis viscosa (Ruiz & Pav.) Kuntze var. nigricans Kuntze, *Molina parviflora* Ruiz & Pav., *Molina salicifolia* Ruiz & Pav., *Molina striata* Ruiz & Pav., *Pingraea marginalis* (DC.) F.H.Hellwig

Shrub.

Departmental distribution: Alto Paraguay, Boquerón, Central, Itapúa, Presidente Hayes.

Voucher: *F. Mereles et al. 9917* (BM, FCQ, G).

***Bidens pilosa*** L. var. ***pilosa***, Sp. Pl. 2: 832. 1753.

Syn.: *Bidens californica* DC., *Bidens hispida* Kunth, *Bidens hirsuta* Nutt., hom. illeg., Bidens leucantha Willd. f. discoidea Sch.Bip., Bidens leucantha var. pilosa (L.) Griseb., Bidens leucantha var. sundaica (Blume) Hassk., *Bidens montaubanii* Phil., Bidens pilosa L. var. brevifoliata Hieron, Bidens pilosa var. discoidea Sch.Bip., Bidens pilosa var. dubia (Cass.) O.E.Schulz, Bidens pilosa var. minor (Blume) Sherff, *Bidens reflexa* Link, *Bidens sundaica* Blume, Bidens sundaica var. minor Blume, *Kerneria dubia* Cass.

Herb.

Departmental distribution: Alto Paraguay, Alto Paraná, Amambay, Caaguazú, Canindeyú, Central, Cordillera, Guairá, Itapúa, Misiones, Paraguarí, Presidente Hayes, San Pedro.

Voucher: *J. De Egea et al. 1236* (FCQ).

***Bidens subalternans*** DC. var. ***subalternans***, Prodr. 5: 600. 1836.

Syn.: *Bidens quadrangularis* DC., *Bidens platensis* Manganaro

Herb.

Departmental distribution: Alto Paraguay, Amambay, Caaguazú, Caazapá, Central, Cordillera, Guairá, Paraguarí.

Voucher: *N. Soria 5736* (FCQ, MO).

***Chrysolaena cognata*** (Less.) Dematteis, Bol. Soc. Argent. Bot. 44(1-2): 157. 2009.

Syn.: *Cacalia cognata* (Less.) Kuntze, *Vernonia cinerascens* Sch.Bip. ex Baker, nom. nud., *Vernonia cognata* Less., *Vernonia senecionea* Mart. ex DC., Vernonia senecionea var. adenocarpa DC., Vernonia senecionea f. calvata Chodat

Herb.

Departmental distribution: Alto Paraná, Amambay, Caaguazú, Canindeyú, Central, Concepción, Cordillera, Guairá, Itapúa, Misiones, Paraguarí, San Pedro.

Voucher: *M. Dematteis et al. 3138* (CTES, FCQ).

***Chrysolaena platensis*** (Spreng.) H.Rob., Proc. Biol. Soc. Washington 101: 957. 1988.

Syn.: *Cacalia platensis* (Spreng.) Kuntze, *Conyza platensis* Spreng., *Vernonia platensis* (Spreng.) Less., Vernonia virens Sch.Bip. ex Baker f. robustior Chodat

Subshrub.

Departmental distribution: Amambay, Concepción, Cordillera, Guairá, Misiones, Paraguarí, San Pedro.

Voucher: *J. De Egea et al. 1335* (BM, CTES, FCQ, G).

***Conyza bonariensis*** (L.) Cronquist var. ***angustifolia*** (Cabrera) Cabrera, Man. Fl. Alrededores Buenos Aires 481. 1953.

Syn.: *Conyza linearis* DC., Erigeron bonariensis L. var. angustifolius Cabrera

Herb.

Departmental distribution: Central, Misiones.

Voucher: *F. Mereles et al. 10176* (FCQ).

***Conyza bonariensis*** (L.) Cronquist var. ***bonariensis***, Bull. Torrey Bot. Club 70: 632. 1943.

Syn.: *Conyza hispida* Kunth, *Conyza plebeja* Phil., *Erigeron bonariensis* L., Erigeron bonariensis f. grisea Chodat & Hassl., *Marsea bonariensis* (L.) V.M.Badillo

Herb.

Departmental distribution: Alto Paraguay, Amambay, Caaguazú, Caazapá, Central, Cordillera, Guairá, Paraguarí, Presidente Hayes.

Voucher: *F. Mereles 6450* (FCQ).

***Conyza sumatrensis*** (Retz.) E.Walker var. ***leiotheca*** (S.F.Blake) Pruski & G.Sancho, Novon 16(1): 98–99, f. 1. 2006.

Syn.: *Conyza bilbaoana* J.Remy, Conyza bonariensis (L.) Cronquist var. leiotheca (S.F.Blake) Cuatrec., *Conyza elata* Kunth & Bouché, *Conyza floribunda* Kunth, Conyza floribunda var. laciniata Cabrera, *Conyza myriocephala* J.Remy, Conyza sumatrensis (Retz.) E.Walker var. floribunda (Kunth) J.B.Marshall, *Erigeron bilbaoanus* (J.Remy) Cabrera, Erigeron bonariensis L. var. floribundum (Kunth) Cuatrec., Erigeron bonariensis f. glabrata Speg., Erigeron bonariensis var. leiothecus S.F.Blake, *Erigeron floribundus* (Kunth) Sch.Bip., Marsea bonariensis (L.) V.M.Badillo var. leiotheca (S.F.Blake) V.M.Badillo

Herb.

Departmental distribution: Alto Paraná, Amambay, Caaguazú, Central, Guairá, Itapúa, Paraguarí.

Voucher: *N. Soria 2684* (FCQ, MO).

***Eclipta prostrata*** (L.) L., Mant. Pl. Altera 286. 1771.

Syn.: *Eclipta alba* (L.) Hassk., *Eclipta erecta* L., nom. illeg., *Galinsoga oblongifolia* (Hook.) DC., *Polygyne inconspicua* Phil., *Verbesina alba* L., *Verbesina conyzoides* Trew, *Verbesina prostrata* L., *Wiborgia oblongifolia* Hook.

Herb.

Departmental distribution: Alto Paraguay, Alto Paraná, Amambay, Boquerón, Canindeyú, Central, Concepción, Cordillera, Guairá, Misiones, Presidente Hayes, San Pedro.

Voucher: *F. Mereles & J. De Egea 10129* (FCQ).

***Emilia fosbergii*** Nicolson, Phytologia 32(1): 34. 1975.

Syn.: *Emilia javanica* auct. non (Burm.) C.B.Rob., *Emilia sagittata* auct. non (Vahl) DC.

Herb. Introduced.

Departmental distribution: Alto Paraná, Amambay, Caaguazú, Canindeyú, Central, Cordillera, Guairá, Misiones, San Pedro.

Voucher: *F. Mereles & J. De Egea 10147* (FCQ).

***Erechtites valerianifolius*** (Link ex Spreng.) DC. f. ***valerianifolius***, Prodr. 6: 295. 1838.

Syn.: *Crassocephalum valerianifolium* (Link ex Spreng.) Less., *Erechtites gardneriana* Cabrera, *Erechtites valerianifolius* (Wolf) DC., nom. inval., *Gynura rosea* Ridl., *Senecio valerianifolius* Wolf ex Link, nom. nud., *Senecio valerianifolius* Desf., hom. illeg., *Senecio valerianifolius* Gardner, hom. illeg., *Senecio valerianifolius* Link ex Spreng., *Sonchus erythropappus* Meyen & Walp. ex Walp.

Herb.

Departmental distribution: Alto Paraná, Amambay, Caaguazú, Caazapá, Canindeyú, Central, Cordillera, Guairá, Itapúa, Paraguarí, San Pedro.

Voucher: *J. De Egea et al. 1237* (FCQ).

***Eupatorium inulifolium*** Kunth, Nov. Gen. Sp. 4(15): 85 (ed. fol.). 1818.

Shrub.

Departmental distribution: Alto Paraná, Amambay, Boquerón, Caaguazú, Caazapá, Canindeyú, Central, Concepción, Cordillera, Guairá, Itapúa, Paraguarí, Presidente Hayes, San Pedro.

Voucher: *F. Mereles 9930* (BM, FCQ, G).

***Flaveria bidentis*** (L.) Kuntze, Revis. Gen. Pl. 3(3): 148. 1898.

Syn.: *Ethulia bidentis* L., *Eupatorium chilense* Molina, *Flaveria bidentis* (L.) B.L.Rob., comb. superfl., *Flaveria bonariensis* DC., *Flaveria capitata* Juss. ex Sm., *Flaveria chilensis* (Molina) J.F.Gmel., *Flaveria contrayerba* (Cav.) Pers., *Milleria chiloensis* Juss., nom. nud., *Milleria contrayerba* Cav., *Vermifuga corymbosa* Ruiz & Pav., nom. illeg.

Herb.

Departmental distribution: Alto Paraguay, Boquerón, Concepción, Presidente Hayes.

Voucher: *F. Mereles 5667* (CTES, FCQ).

***Galinsoga parviflora*** Cav., Icon. 3(2): 41–42, pl. 281. 1796.

Syn.: *Adventina parviflora* Raf., *Galinsoga quinqueradiata* Ruiz & Pav., nom. superfl., *Wiborgia acmella* Roth, *Wiborgia parviflora* (Cav.) Kunth

Herb.

Departmental distribution: Amambay, Central, Guairá.

Voucher: *G. Céspedes 422* (FCQ).

***Gamochaeta americana*** (Mill.) Wedd., Chlor. And. 1: 151. 1856.

Syn.: Gamochaeta americana var. alpina Wedd., *Gamochaeta guatemalensis* (Gand.) Cabrera, *Gnaphalium americanum* Mill., *Gnaphalium consanguineum* Gaudich., *Gnaphalium guatemalense* Gand., Gnaphalium purpureum L. var. americaum (Mill.) Klatt.

Herb.

Departmental distribution: Alto Paraná, Amambay, Central, Cordillera, Presidente Hayes.

Voucher: *G. Céspedes 432* (FCQ).

***Gamochaeta calviceps*** (Fernald) Cabrera, Bol. Soc. Argent. Bot. 9: 368. 1961.

Syn.: *Gnaphalium calviceps* Fernald

Herb.

Departmental distribution: Alto Paraguay, Amambay, Caaguazú, Central, Concepción, Cordillera, Paraguarí, San Pedro.

Voucher: *J. De Egea et al. 1227* (BM, FCQ).

***Gamochaeta coarctata*** (Willd.) Kerguélen, Lejeunia 120: 104. 1987.

Syn.: *Gamochaeta spicata* Cabrera, *Gnaphalium coarctatum* Willd., Gnaphalium purpureum L. var. spicatum Baker, nom. illeg., *Gnaphalium spicatum* Lam., nom. illeg.

Herb.

Departmental distribution: Alto Paraguay, Alto Paraná, Caazapá, Central, Cordillera, Guairá, Itapúa, San Pedro.

Voucher: *J. De Egea et al. 1322* (FCQ).

***Gamochaeta pensylvanica*** (Willd.) Cabrera, Bol. Soc. Argent. Bot. 9: 375. 1961.

Syn.: *Gamochaeta platensis* (Cabrera) Cabrera, *Gnaphalium pensylvanicum* Willd., *Gnaphalium peregrinum* Fernald, *Gnaphalium platense* Cabrera, Gnaphalium purpureum L. ssp. pensylvanica (Willd.) O.Bolòs & Vigo, Gnaphalium purpureum var. spathulatum Baker, *Gnaphalium spathulatum* Lam., hom. illeg.

Herb.

Departmental distribution: Alto Paraguay, Alto Paraná, Boquerón, Canindeyú, Central, Cordillera, Guairá, Paraguarí, Presidente Hayes.

Voucher: *I. Basualdo 395* (FCQ).

***Lepidaploa remotiflora*** (Rich.) H.Rob., Proc. Biol. Soc. Washington 103: 491. 1990.

Syn.: *Vernonia hirtiflora* Sch.Bip. ex Baker, *Vernonia lithospermoides* Baker, *Vernonia remotiflora* Rich., Vernonia remotiflora var. tricholepis (DC.) Baker, *Vernonia sessiliflora* Willd. ex Less., *Vernonia tricholepis* DC.

Herb.

Departmental distribution: Alto Paraguay, Alto Paraná, Amambay, Caaguazú, Caazapá, Canindeyú, Central, Concepción, Cordillera, Guairá, Itapúa, Misiones, Ñeembucú, Paraguarí, Presidente Hayes, San Pedro.

Voucher: *J. De Egea et al. 1223* (BM, FCQ).

***Parthenium hysterophorus*** L., Sp. Pl. 2: 988. 1753.

Syn.: *Argyrochaeta bipinnatifida* Cav., *Echetrosis pentasperma* Phil., *Parthenium glomeratum* Rollins, *Parthenium lobatum* Buckley, *Parthenium pinnatifidum* Stokes, nom. superfl., *Villanova bipinnatifida* Ortega

Herb.

Departmental distribution: Alto Paraguay, Amambay, Presidente Hayes.

Voucher: *J. De Egea et al. 142* (BM, CTES, FCQ, G, MO).

***Pluchea sagittalis*** (Lam.) Cabrera, Bol. Soc. Argent. Bot. 3: 36. 1949.

Syn.: *Conyza sagittalis* Lam., *Epaltes brasiliensis* DC., *Gnaphalium suaveolens* Vell., *Pluchea quitoc* DC., *Pluchea suaveolens* (Vell.) Kuntze

Herb.

Departmental distribution: Alto Paraguay, Amambay, Boquerón, Caaguazú, Caazapá, Central, Concepción, Cordillera, Guairá, Paraguarí, Presidente Hayes.

Voucher: *F. Mereles et al. 10048* (FCQ, G).

***Praxelis clematidea*** (Griseb.) R.M.King & H.Rob., Phytologia 20: 194. 1970.

Syn.: *Eupatorium catarium* Veldkamp, *Eupatorium clematideum* Griseb., hom. illeg., *Eupatorium pauciflorum* auct. non Kunth, *Eupatorium urticifolium* auct. non L., Eupatorium urticifolium L. f. var. clematideum Hieron. ex Kuntze, Eupatorium urticifolium f. var. nanum Hieron. ex Kuntze

Herb.

Departmental distribution: Alto Paraguay, Amambay, Boquerón, Caaguazú, Canindeyú, Central, Concepción, Cordillera, Guairá, Misiones, Ñeembucú, Paraguarí, Presidente Hayes, San Pedro.

Voucher: *W.J. Hahn 1613* (MO, PY).

***Senecio brasiliensis*** (Spreng.) Less var. ***brasiliensis***, Linnaea 6: 249. 1831.

Syn.: *Cineraria brasiliensis* Spreng.

Herb.

Departmental distribution: Alto Paraná, Amambay, Caaguazú, Caazapá, Canindeyú, Itapúa, Misiones.

Voucher: *N. Soria 2513* (CTES, MO).

***Senecio grisebachii*** Baker var. ***grisebachii***, Fl. Bras. 6(3): 313. 1884.

Herb.

Departmental distribution: Alto Paraguay, Amambay, Caaguazú, Caazapá, Canindeyú, Central, Cordillera, Guairá, Itapúa, Misiones, Ñeembucú, Paraguarí, Presidente Hayes, San Pedro.

Voucher: *F. Mereles et al. 10005* (FCQ).

***Senecio hieronymi*** Griseb., Abh. Königl. Ges. Wiss. Göttingen 24: 205. 1879.

Syn.: *Senecio charaguensis* Cuatrec., *Senecio tafiensis* Cabrera

Subshrub.

Departmental distribution: Alto Paraná, Canindeyú, Central, Guairá, Paraguarí.

Voucher: *J. De Egea et al. 1331* (BM, FCQ).

***Solidago chilensis*** Meyen var. ***chilensis***, Reise Erde 1: 311. 1834.

Syn.: *Aster sagei* Phil., *Solidago coquimbana* Phil., *Solidago floribunda* Phil., *Solidago laxiflora* Phil., *Solidago linearifolia* DC., Solidago linearifolia var. brachypoda Speg., Solidago microglossa DC. var. linearifolia (DC.) Baker, *Solidago parviflora* Phil., *Solidago recta* Phil., *Solidago valdiviana* Phil.

Herb.

Departmental distribution: Alto Paraguay, Boquerón, Cordillera.

Voucher: *I. Basualdo 111* (FCQ).

***Solidago microglossa*** DC., Prodr. 5: 332. 1836.

Syn.: Solidago chilensis Meyen var. megapotamica (DC.) Cabrera, Solidago microglossa DC. var. megapotamica DC.

Herb.

Departmental distribution: Alto Paraguay, Alto Paraná, Amambay, Caaguazú, Caazapá, Central, Concepción, Cordillera, Guairá, Paraguarí, Presidente Hayes, San Pedro.

Voucher: *M. Ortíz 1004* (FCQ).

***Soliva sessilis*** Ruiz & Pav., Syst. Veg. Fl. Peruv. Chil. 1: 215. 1798.

Syn.: *Gymnostyles alata* Spreng., *Gymnostyles barclayana* (DC.) Steud., *Gymnostyles chilensis* Spreng., *Gymnostyles pterosperma* Juss., *Soliva alata* (Spreng.) DC., *Soliva barclayana* DC., *Soliva daucifolia* Nutt., *Soliva microloma* Phil., *Soliva neglecta* Cabrera, *Soliva pterosperma* (Juss.) Less., Soliva sessilis Ruiz & Pav. var. barclayana (DC.) Baker, *Soliva valdiviana* Phil.

Herb.

Departmental distribution: Alto Paraná, Concepción, Cordillera, Guairá, Misiones, Paraguarí, San Pedro.

Voucher: *L. Pérez 448* (FCQ).

***Sonchus oleraceus*** L., Sp. Pl. 2: 794. 1753.

Syn.: *Sonchus gracilis* Phil., *Sonchus rivularis* Phil.

Herb. Introduced.

Departmental distribution: Alto Paraguay, Alto Paraná, Boquerón, Caazapá, Canindeyú, Central, Guairá, San Pedro.

Voucher: *J. De Egea et al. 1238* (BM, FCQ).

***Synedrellopsis grisebachii*** Hieron. & Kuntze, Revis. Gen. Pl. 3(3): 180. 1898.

Syn.: *Synedrella nodiflora* Griseb. ex Kuntze, nom. nud.

Herb.

Departmental distribution: Alto Paraguay, Amambay, Boquerón, Central, Itapúa, San Pedro.

Voucher: *J. De Egea & F. Mereles 1357* (BM, CTES, FCQ, G).

***Tagetes minuta*** L., Sp. Pl. 2: 887. 1753.

Syn.: *Tagetes bonariensis* Pers., *Tagetes glandulifera* Schrank, *Tagetes glandulosa* Link, *Tagetes porophyllum* Vell.

Herb.

Departmental distribution: Alto Paraguay, Amambay, Central, Cordillera, Paraguarí, Presidente Hayes.

Voucher: *A. Schinini 2401* (FCQ).

***Taraxacum officinale*** F.H.Wigg., Prim. Fl. Holsat. 56. 1780.

Syn.: *Leontodon taraxacum* L., *Leontodon vulgaris* Lam., nom. illeg., *Taraxacum dens-leonis* Desf., *Taraxacum subspathulatum* A.J.Richards, *Taraxacum vulgare* (Lam.) Schrank., nom. illeg.

Herb. Introduced.

Departmental distribution: Alto Paraná, Caazapá, Guairá.

Voucher: *R. Degen 982* (FCQ).

*Tridax procumbens L., Sp. Pl. 2: 900. 1753.

Syn.: *Amellus pedunculatus* Ortega ex Willd., nom. inval., *Balbisia canescens* Pers., *Balbisia divaricata* Cass., *Balbisia elongata* Willd., *Balbisia pedunculata* Ortega ex Hoffmans., nom. illeg., Tridax procumbens L. var. canescens (Pers.) DC., Tridax procumbens var. ovatifolia B.L.Rob. ex Greenm.

Herb.

Departmental distribution: Boquerón, Central, Concepción, San Pedro.

Voucher: *F. Mereles & S. Soria 9944* (BM, FCQ, G).

***Vernonanthura tweedieana*** (Baker) H.Rob., Phytologia 73: 74. 1992.

Syn.: *Chrysocoma arborea* Vell., *Vernonia tweedieana* Baker

Shrub.

Departmental distribution: Alto Paraguay, Alto Paraná, Amambay, Caaguazú, Central, Cordillera, Guairá, Itapúa, Misiones, Paraguarí, Presidente Hayes, San Pedro.

Voucher: *F. Mereles et al. 9907* (FCQ, G).

***Vernonia incana*** Less., Linnaea 4: 277. 1829.

Syn.: *Cacalia incana* (Less.) Kuntze, *Vernonia inmunis* Griseb.

Herb.

Departmental distribution: Alto Paraná, Amambay, Central, Ñeembucú, Paraguarí, Presidente Hayes.

Voucher: *M. Ortíz 551* (FCQ).

#### BORAGINACEAE

***Heliotropium leiocarpum*** Morong, Ann. New York Acad. Sci. 7: 168. 1893.

Syn.: Heliotropium leiocarpum Morong f. albiflora Chodat, Heliotropium leiocarpum f. minor Chodat

Subshrub.

Departmental distribution: Alto Paraná, Central, Cordillera, Paraguarí, Presidente Hayes.

Voucher: *A. Krapovickas & C.L. Cristóbal 44544* (FCQ).

#### BRASSICACEAE

***Brassica rapa*** L., Sp. Pl. 2: 666. 1753.

Syn.: *Brassica campestris* L.

Herb. Introduced.

Departmental distribution: Alto Paraguay, Boquerón, Central.

Voucher: *M. Ortíz 283* (FCQ).

*Capsella bursa-pastoris (L.) Medik., Pfl.-Gatt. 85. 1792.

Syn.: *Thlaspi bursa-pastoris* L.

Herb. Introduced.

Departmental distribution: Itapúa.

Voucher: *E. Lurvey 181* (Lurvey 1983).

***Lepidium didymum*** L., Syst. Nat., ed. 12. 2: 433. 1767.

Syn.: *Coronopus didymus* (L.) Sm., Coronopus didymus var. macrocarpus Muschl., Coronopus didymus var. procumbens Muschl., *Coronopus leptocarpus* Boelcke, Coronopus leptocarpus var. microcarpus Boelcke, *Lepicochlea americana* Rojas Acosta, *Senebiera didyma* (L.) Pers., *Senebiera pinnatifica* DC.

Herb.

Departmental distribution: Alto Paraná, Amambay, Caazapá, Central, Cordillera.

Voucher: *I. Basualdo 5223* (FCQ).

***Lepidium virginicum*** L., Sp. Pl. 2: 645. 1753.

Herb. Introduced.

Departmental distribution: Amambay, Caaguazú, Central, Guairá, Itapúa.

Voucher: *E. Lurvey 359* (Lurvey 1983).

***Raphanus raphanistrum*** L., Sp. Pl. 2: 669. 1753.

Herb. Introduced.

Departmental distribution: Amambay, Cordillera, Itapúa.

Voucher: *E. Lurvey 374* (Lurvey 1983).

***Raphanus sativus*** L., Sp. Pl. 2: 669. 1753.

Herb. Introduced.

Departmental distribution: Amambay, Central, Cordillera, Misiones, San Pedro.

Voucher: *J. De Egea et al. 1268* (FCQ).

#### CAMPANULACEAE

***Lobelia xalapensis*** Kunth, Nov. Gen. Sp. 3: 315. 1819.

Syn.: Dortmannia cliffortiana Kuntze var. xalapensis (Kunth) Kuntze, *Dortmannia mollis* (Graham) Kuntze, *Dortmannia monticola* (Kunth) Kuntze, *Dortmannia ocimoides* (Kunze) Kuntze, *Lobelia cliffortiana* L., Lobelia cliffortiana var. xalapensis (Kunth) A.Gray, *Lobelia mollis* Graham, *Lobelia monticola* Kunth, *Lobelia ocimoides* Kunze, *Lobelia palmaris* Willd. ex Roem. & Schult., *Rapuntium monticulum* (Kunth) C.Presl, *Rapuntium xalapense* (Kunth) C.Presl

Herb.

Departmental distribution: Alto Paraguay, Amambay, Boquerón, Caazapá, Canindeyú, Central, Concepción, Cordillera, Guairá, Paraguarí, San Pedro.

Voucher: *J. De Egea et al. 1317* (BM, FCQ).

***Triodanis perfoliata*** (L.) Nieuwl. subsp. ***biflora*** (Ruiz & Pav.) Lammers, Novon 16(1): 72. 2006.

Syn.: *Campanula biflora* Ruiz & Pav., *Campanula ludoviciana* Torr. ex A.Gray, nom. nud., *Campanula montevidensis* Spreng., *Dysmicodon californicum* Nutt., *Dysmicodon ovatum* Nutt., *Legouzia biflora* (Ruiz & Pav.) Britton, *Pentagonia biflora* (Ruiz & Pav.) Kuntze, *Specularia biflora* (Ruiz & Pav.) Fisch. & A.Gray, *Specularia californica* (Nutt.) Vatke, *Specularia ovata* (Nutt.) Vatke, *Specularia perfoliata* (L.) A.DC., Specularia perfoliata f. ramosa Arechav., Specularia perfoliata f. rigida Arechav., *Triodanis biflora* (Ruiz & Pav.) Greene, Triodanis perfoliata (L.) Nieuwl. var. biflora (Ruiz & Pav.) T.R.Bradley

Herb.

Departmental distribution: Alto Paraná, Central, Guairá, Paraguarí.

Voucher: *J. De Egea et al. 1313* (FCQ).

***Wahlenbergia linarioides*** (Lam.) A.DC., Monogr. Campan. 158. 1830.

Syn.: *Campanula arida* Kunth, *Campanula chilensis* Molina, *Campanula linarioides* Lam., *Wahlenbergia arida* (Kunth) Griseb., Wahlenbergia linarioides (Lam.) A.DC. var. arida (Kunth) A.DC., Wahlenbergia linarioides var. micrantha Phil.

Herb.

Departmental distribution: Alto Paraná, Amambay, Caaguazú, Caazapá, Canindeyú, Central, Guairá, Itapúa, Ñeembucú, Paraguarí.

Voucher: *F. Mereles et al. 10088* (FCQ, G).

#### CARYOPHYLLACEAE

***Cerastium rivulariastrum*** Möschl & Pedersen, Darwiniana 16(1-2): 118. 1970.

Herb.

Departmental distribution: Canindeyú, Central, Guairá, Misiones, Paraguarí.

Voucher: *G. Céspedes 435* (FCQ).

***Drymaria cordata*** (L.) Willd. ex Roem. & Schult., Syst. Veg. (ed. 15 bis) 5: 406. 1819.

Syn.: Drymaria cordata (L.) Willd. ex Roem. & Schult. var. pacifica Mizush., *Holosteum cordatum* L.

Herb.

Departmental distribution: Central, Cordillera, Guairá, Paraguarí.

Voucher: *E. Zardini & R. Velázquez 27329* (FCQ, MO).

***Paronychia communis*** Cambess. var. ***communis***, Fl. Bras. Merid. (quarto ed.) 2(15): 186. 1829.

Herb.

Departmental distribution: Alto Paraná, Canindeyú, Cordillera, Paraguarí.

Voucher: *F. Mereles et al. 10073* (FCQ, G).

***Stellaria media*** (L.) Cirillo var. ***gymnocalyx*** Trautv., Acta Horti Petrop. 1: 33. 1881.

Syn.: *Stellaria glabra* Raunk.

Herb. Introduced.

Departmental distribution: Itapúa.

Voucher: *E. Lurvey 205* (CTES).

#### CLEOMACEAE

***Cleome aculeata*** L. var. ***aculeata***, Syst. Nat., ed. 12. 3: 232. 1768.

Herb.

Departmental distribution: Alto Paraguay, Central, Concepción, Cordillera, Guairá, Itapúa, Paraguarí, Presidente Hayes.

Voucher: *M. Ortíz 317* (FCQ).

***Cleome tucumanensis*** Iltis, Brittonia 12: 284. 1960.

Syn.: *Cleome flexuosa* Griseb., nom. illeg.

Herb.

Departmental distribution: Alto Paraguay, Boquerón, Central, Presidente Hayes.

Voucher: *F. Mereles & S. Soria 9941* (FCQ, G).

#### COMMELINACEAE

*Commelina benghalensis L., Sp. Pl. 1: 41. 1753.

Herb. Introduced.

Departmental distribution: Cordillera, Guairá, Paraguarí, San Pedro.

Voucher: *J. De Egea et al. 1273* (FCQ).

***Commelina diffusa*** Burm.f. var. ***diffusa***, Fl. Indica 18, pl. 7, f. 2. 1768.

Syn.: *Commelina cayennensis* Rich., Commelina cayennensis var. pubescens Griseb., *Commelina longicaulis* Jacq., *Commelina nudiflora* auct. non L.

Herb.

Departmental distribution: Concepción, Guairá, Misiones, Ñeembucú, Paraguarí.

Voucher: *F. Mereles & J. De Egea 10149* (FCQ, G).

***Commelina diffusa*** Burm.f. var. ***gigas*** (Small) Faden, Ann. Missouri Bot. Gard. 80: 213. 1993.

Syn.: *Commelina gigas* Small

Herb.

Departmental distribution: Boquerón, Central, Cordillera, Guairá, Itapúa, Paraguarí.

Voucher: *F. Mereles et al. 9919* (FCQ, G).

***Commelina erecta*** L. var. ***angustifolia*** (Michx.) Fernald, Rhodora 42: 439. 1940.

Syn.: *Commelina angustifolia* Michx., Commelina virginica L. var. angustifolia (Michx.) C.B.Clarke

Herb.

Departmental distribution: Boquerón, Cordillera.

Voucher: *F. Mereles & S. Soria 9964* (FCQ, G).

***Commelina erecta*** L. var. ***erecta***, Sp. Pl. 1: 41. 1753.

Syn.: *Commelina elegans* Kunth, *Commelina pohliana* Seub., *Commelina sulcata* Willd., *Commelina virginica* auct. non L., Commelina virginica L. var. australis C.B.Clarke

Herb.

Departmental distribution: Alto Paraguay, Amambay, Canindeyú, Central, Ñeembucú, Paraguarí, Presidente Hayes, San Pedro.

Voucher: *F. Mereles 9936* (BM, FCQ, G).

***Commelina platyphylla*** Klotzsch, Reis. Br.-Guiana 3: 897. 1849.

Syn.: *Commelina balansae* (C.B.Clarke) Herter, Commelina platyphylla var. balansae C.B.Clarke

Herb.

Departmental distribution: Boquerón, Caaguazú, Central, Concepción, Cordillera, Itapúa, Misiones, Ñeembucú, Paraguarí, Presidente Hayes.

Voucher: *F. Mereles & J. De Egea 10127* (FCQ).

#### CONVOLVULACEAE

***Ipomoea cordatotriloba*** Dennst. var. ***australis*** (O’Donell) D.F.Austin, Taxon 37(1): 185. 1988.

Syn.: *Ipomoea trichocarpa* Elliot, nom. illeg., Ipomoea trichocarpa Elliott var. australis O’Donell,

Vine.

Departmental distribution: Amambay, Caaguazú, Central, Cordillera, Guairá, Paraguarí, San Pedro.

Voucher: *E. Zardini 9984* (FCQ, MO).

***Ipomoea grandifolia*** (Dammer) O’Donell, Arq. Mus. Paraense 9: 222. 1952.

Syn.: *Ipomoea triloba* auct. non L., *Jacquemontia grandifolia* Dammer

Vine.

Departmental distribution: Alto Paraguay, Caazapá, Canindeyú, Central, Concepción, Cordillera, Guairá, Misiones, Ñeembucú, Paraguarí, San Pedro.

Voucher: *F. Mereles & J. De Egea 10140* (FCQ, G).

***Ipomoea nil*** (L.) Roth, Catal. Bot. 1: 36. 1797.

Syn.: *Convolvulus nil* L., *Convolvulus tomentosus* Vell., *Ipomoea cuspidata* Ruiz & Pav., *Ipomoea longicuspis* Meisn., nom. illeg., *Ipomoea scabra* Forssk., *Pharbitis cuspidata* (Ruiz & Pav.) G.Don

Vine.

Departmental distribution: Boquerón, Caazapá, Canindeyú, Central, Cordillera, Guairá, Itapúa, Paraguarí, Presidente Hayes, San Pedro.

Voucher: *J. De Egea et al. 1318* (BM, FCQ, G).

#### CUCURBITACEAE

***Cucurbitella asperata*** (Gillies ex Hook. & Arn.) Walp., Repert. Bot. Syst. 6: 50. 1846.

Syn.: *Cucurbita asperata* Gillies ex Hook. & Arn., *Cucurbita urkupinana* Cárdenas, *Cucurbitella cucumifolia* (Griseb.) Cogn., *Cucurbitella duriaei* (Naudin) Cogn., *Cucurbitella integrifolia* Cogn., Cucurbitella integrifolia Cogn. var. glabrior Cogn, *Cucurbitella urkupinana* (Cárdenas) C.Jeffrey, *Prasopepon cucumifolius* Griseb., *Prasopepon durieui* Naudin, *Schizostigma asperatum* (Gillies ex Hook. & Arn.) Arn., comb. illeg.

Vine.

Departmental distribution: Alto Paraguay, Boquerón, Central, Concepción, Paraguarí, Presidente Hayes, San Pedro.

Voucher: *J. De Egea et al. 1226* (BM, FCQ).

#### CYPERACEAE

***Bulbostylis capillaris*** (L.) Kunth ex C.B.Clarke var. ***capillaris***, Fl. Brit. India 6(19): 652. 1893.

Syn.: *Abildgaardia capillaris* (L.) Lye, *Fimbristylis capillaris* (L.) A.Gray, *Isolepis capillaris* (L.) Roem. & Schult., *Scirpus capillaris* L., *Stenophyllus capillaris* (L.) Britton

Herb.

Departmental distribution: Alto Paraguay, Caaguazú, Caazapá, Guairá, Paraguarí.

Voucher: *J. De Egea et al. 1327* (FCQ).

***Cyperus aggregatus*** Endl., Cat. Horti Vindob. 1: 93. 1842.

Syn.: Cyperus aggregatus (Willd.) Endl. var. gigas (Lindm.) Guagl., *Cyperus argentinus* Boeck., *Cyperus cayennensis* (Lam.) Britton, nom. illeg., Cyperus cayennensis var. gigas (Lindm.) Barros, Cyperus cayennensis var. umbellato-flavus (C.B.Clarke) Barros, *Cyperus flavomariscus* Griseb., *Cyperus flavus* (Vahl) Nees, nom. illeg., Cyperus flavus var. aggregatus (Willd.) Kük., Cyperus flavus var. angustatus Kük., Cyperus flavus var. argentinus (Boeck.) Kük. ex Osten, Cyperus flavus var. gigas (Lindm.) Kük., Cyperus retrorsus Chapm. var. australis (Lindm.) Kük., *Didymia cyperomorpha* Phil., *Kyllinga cayennensis* Lam., *Mariscus aggregatus* Willd., *Mariscus cayennensis* (Lam.) Urb., Mariscus cylindricus Elliot var. australis Lindm., *Mariscus flavus* Vahl, Mariscus flavus var. gigas Lindm., Cyperus flavus var. laevis (Kunth) Kük., Mariscus flavus f. umbellato-flava C.B.Clarke

Herb.

Departmental distribution: Alto Paraguay, Alto Paraná, Amambay, Canindeyú, Central, Cordillera, Guairá, Itapúa, Misiones, Ñeembucú, Paraguarí, Presidente Hayes, San Pedro.

Voucher: *J. De Egea et al. 1289* (BM, FCQ).

►***Cyperus eragrostis*** Lam. var. ***eragrostis***, Tabl. Encycl. i. 196. 1791.

Syn.: *Cyperus compressus* Jacq., *Cyperus declinatus* Moench, Cyperus eragrostis Lam. f. latifrons Kük., Cyperus eragrostis f. tener Kük., *Cyperus monandrus* Roth, *Cyperus vegetus* Willd., Cyperus vegetus var. obtusangulus Kuntze

Herb.

Departmental distribution: Guairá.

Voucher: *F. Mereles & S. Soria 9949* (FCQ, G).

***Cyperus esculentus*** L. var. ***leptostachyus*** Boeck., Linnaea 36: 290. 1870.

Syn.: *Chlorocyperus phymatodes* (Muhl.) Palla, Cyperus esculentus L. var. phymatodes (Muhl.) Kük., *Cyperus phymatodes* Muhl.

Herb. Introduced.

Departmental distribution: Alto Paraguay, Alto Paraná, Central, Cordillera, Itapúa.

Voucher: *J. De Egea et al. 325* (BM, MO).

***Cyperus haspan*** L. var. ***haspan***, Sp. Pl. 1: 45. 1753.

Syn.: *Cyperus adenophorus* Schrad., *Cyperus americanus* (Boeck.) Palla, *Cyperus autummalis* (Rottb.) Vahl, *Cyperus cayennensis* Willd. ex Link, *Cyperus efoliatus* Boeck., Cyperus haspan L. var. adenophorus (Schrad.) Kük., Cyperus haspan var. americanus Boeck., Cyperus haspan ssp. juncoides (Lam.) Kük., Cyperus haspan var. riparius (Schrad. ex Nees) Kük., *Cyperus juncoides* Lam., *Cyperus riparius* Schrad. ex Nees, *Scirpus autumnalis* Rottb., hom. illeg.

Herb.

Departmental distribution: Alto Paraguay, Caaguazú, Caazapá, Canindeyú, Central, Cordillera, Guairá, Itapúa, Misiones, Ñeembucú, Paraguarí.

Voucher: *F. Mereles & S. Soria 10201* (FCQ).

***Cyperus odoratus*** L., Sp. Pl. 1: 46. 1753.

Syn.: *Cyperus acicularis* (Nees) Steud., *Cyperus cephalophorus* J.Presl & C.Presl, *Cyperus conglobatus* Link, *Cyperus densiflorus* G.Mey., *Cyperus engelmannii* Steud., *Cyperus ferax* Rich., Cyperus ferax var. acicularis (Nees) Kük., Cyperus ferax var. bulbiferus Barros, Cyperus ferax var. conglobatus (Link) Kük., Cyperus ferax ssp. engelmanni (Steud.) Kük., Cyperus ferax var. maximilianii (Schrad. ex Nees) Boeck., Cyperus ferax ssp. speciosus (Vahl) Kük., *Cyperus ferox* Vahl, *Cyperus flexuosus* Vahl, *Cyperus hamiltonii* Kunth, *Cyperus huarmensis* (Kunth) M.C.Johnst., *Cyperus jubaeflorus* Rudge, *Cyperus maximilianii* (Schrad. ex Nees) Griseb., *Cyperus pohlianus* (Nees) Kuntze, *Cyperus speciosus* Vahl, *Diclidium lenticulare* Schrad. ex Nees, *Diclidium lomentaceum* Nees, *Diclidium maximilianii* Schrad. ex Nees, *Diclidium odoratum* Schrad. ex Nees, *Diclidium uliginosum* Schrad. ex Nees, *Mariscus ferax* (Rich.) C.B.Clarke, *Mariscus pohlianus* Nees, *Torulinium confertum* Desv. ex Ham., *Torulinium ferax* (Rich.) Urb., *Torulinium odoratum* (L.) S.S.Hooper

Herb.

Departmental distribution: Alto Paraguay, Alto Paraná, Amambay, Boquerón, Caaguazú, Caazapá, Central, Concepción, Cordillera, Guairá, Itapúa, Misiones, Ñeembucú, Paraguarí, Presidente Hayes, San Pedro.

Voucher: *F. Mereles & S. Soria 9965* (FCQ, G).

***Cyperus rotundus*** L., Sp. Pl. 1: 45. 1753.

Syn.: *Chlorocyperus rotundus* (L.) Palla

Herb.

Departmental distribution: Caazapá, Cordillera, Itapúa.

Voucher: *F. Mereles et al. 9920* (FCQ).

***Cyperus tenuis*** Sw., Prodr. 20. 1788.

Syn.: *Cyperus caracasanus* Kunth

Herb.

Departmental distribution: Caaguazú, Misiones, Paraguarí.

Voucher: *F. Mereles & J. De Egea 10136* (FCQ).

***Eleocharis montana*** (Kunth) Roem. & Schult., Syst. Veg. 2: 153. 1817.

Syn.: *Eleocharis consanguinea* Kunth, Eleocharis montana var. nodulosa (Roth) Svenson, *Eleocharis nodulosa* (Roth) Schult., Eleocharis nodulosa f. trigyna Barros, *Eleogenus nodulosus* (Roth) Nees, *Scirpus montanus* Kunth, *Scirpus nodulosus* Roth

Herb aquatic.

Departmental distribution: Alto Paraguay, Alto Paraná, Amambay, Caaguazú, Caazapá, Canindeyú, Central, Concepción, Cordillera, Guairá, Itapúa, Misiones, Ñeembucú, Paraguarí, Presidente Hayes, San Pedro.

Voucher: *G. Céspedes & al 443* (BM, FCQ).

***Rhynchospora corymbosa*** (L.) Britton var. ***corymbosa***, Trans. New York Acad. Sci. 11: 84. 1892.

Syn.: *Calyptrostylis fascicularis* Nees, *Calyptrostylis florida* (Rudge) Nees, *Dichromena corymbosa* (L.) J.F.Macbr., *Rhynchospora aurea* Vahl, Rhynchospora corymbosa (L.) Britton var. florida (Rudge) Kük., *Rhynchospora florida* (Rudge) Schult., *Rhynchospora surinamensis* (Rottb.) Nees, *Schoenus floridus* Rudge, *Schoenus surinamensis* Rottb., *Scirpus corymbosus* L.

Herb.

Departmental distribution: Alto Paraguay, Amambay, Caaguazú, Canindeyú, Central, Concepción, Cordillera, Guairá, Itapúa, Misiones, Ñeembucú, Paraguarí, Presidente Hayes, San Pedro.

Voucher: *G. Céspedes et al. 444* (FCQ).

#### EUPHORBIACEAE

***Acalypha communis*** Müll.Arg., Linnaea 34: 23. 1865.

Syn.: *Acalypha agrestis* Morong ex Britton, *Acalypha apicalis* N.E.Br., Acalypha communis Müll.Arg. var. agrestis (Morong ex Britton) Chodat, Acalypha communis f. grandifolia Chodat & Hassl., Acalypha communis var. guaranitica Chodat & Hassl., Acalypha communis var. hirta (Spreng.) Müll.Arg., comb. illeg., Acalypha communis var. salicifolia Pax & K.Hoffm., Acalypha communis var. saltensis Pax & K.Hoffm., Acalypha communis var. tomentella Müll.Arg., Acalypha communis var. tomentosa Müll.Arg., *Acalypha cordobensis* Müll.Arg., Acalypha cordobensis var. rotundata Griseb., *Acalypha gracilis* Griseb., hom. illeg., *Acalypha hirta* Spreng., hom. illeg., *Acalypha montevidensis* Klotzsch ex Pax & K.Hoffm., nom. nud., *Acalypha paraguariensis* Chodat & Hassl., Acalypha variabilis Klotzsch ex Baill. var. angustifolia Baill., *Ricinocarpus cordobensis* (Müll.Arg.) Kuntze.

Herb or subshrub.

Departmental distribution: Alto Paraguay, Alto Paraná, Amambay, Boquerón, Caazapá, Canindeyú, Central, Concepción, Cordillera, Guairá, Itapúa, Ñeembucú, Paraguarí, Presidente Hayes, San Pedro.

Voucher: *F. Mereles 9929* (BM, CTES, FCQ, G).

***Cnidoscolus albomaculatus*** (Pax) I.M.Johnst., Contr. Gray Herb. 68: 86. 1923.

Syn.: *Jatropha albomaculata* Pax, Jatropha albomaculata var. nana (Chodat & Hassl.) Pax, Jatropha albomaculata var. stimulosissima (Chodat & Hassl.) Pax, Jatropha albomaculata var. subcuneata Pax, Jatropha vitifolia Mill. f. nana Chodat & Hassl., Jatropha vitifolia f. stimulosissima Chodat & Hassl.

Herb or shrub.

Departmental distribution: Alto Paraguay, Amambay, Boquerón, Caazapá, Canindeyú, Central, Concepción, Cordillera, Guairá, Itapúa, Ñeembucú, Paraguarí, Presidente Hayes, San Pedro.

Voucher: *F. Mereles & S. Soria 10181* (FCQ).

***Croton glandulosus*** L., Syst. Nat., ed. 10. 2: 1275. 1759.

Syn.: *Croton divaricatus* Sw., Croton glandulosus L. var. scordioides (Lam.) Müll.Arg., *Croton scordioides* Lam.

Herb.

Departmental distribution: Alto Paraná, Amambay, Boquerón, Caazapá, Canindeyú, Central, Concepción, Cordillera, Guairá, Itapúa, Paraguarí.

Voucher: *J. De Egea et al. 1372* (FCQ).

***Euphorbia heterophylla*** L., Sp. Pl. 1: 453. 1753.

Syn.: *Poinsettia heterophylla* (L.) Klotzsch & Garcke

Herb.

Departmental distribution: Alto Paraná, Amambay, Caazapá, Canindeyú, Central, Concepción, Cordillera, Guairá, Itapúa, Paraguarí.

Voucher: *F. Mereles et al. 9921* (BM, FCQ, G).

***Euphorbia hirta*** L. var. ***hirta***, Sp. Pl. 1: 454. 1753.

Syn.: *Chamaesyce hirta* (L.) Millsp., *Euphorbia pilulifera* auct. non L., Euphorbia pilulifera L. var. guaranitica Chodat & Hassl.

Herb. Introduced.

Departmental distribution: Alto Paraguay, Alto Paraná, Central, Itapúa, Paraguarí.

Voucher: *F. Mereles et al. 10096* (FCQ).

***Euphorbia hypericifolia*** L., Sp. Pl. 1: 454. 1753.

Syn.: *Chamaesyce hypericifolia* (L.) Millsp., *Euphorbia boliviana* Rusby

Herb.

Departmental distribution: Alto Paraguay, Itapúa.

Voucher: *F. Mereles et al. 9923* (FCQ, G).

***Euphorbia prostrata*** Aiton, Hort. Kew. 2: 139. 1789.

Syn.: *Chamaesyce prostrata* (Aiton) Small

Herb.

Departmental distribution: Central, Guairá.

Voucher: *F. Mereles 10123* (FCQ).

***Euphorbia selloi*** (Klotzsch & Garcke) Boiss., Prodr. 15(2.1): 50. 1862.

Syn.: *Anisophyllum selloi* Klotzsch & Garcke, *Chamaesyce selloi* (Klotzsch & Garcke) Croizat, Chamaesyce selloi var. brevisemina Croizat, *Euphorbia hassleriana* Chodat, *Euphorbia hebegyne* Pax & K.Hoffm. ex Emrich, Euphorbia selloi var. setosa auct. non Boiss., *Euphorbia setosa* (Boiss.) Müll.Arg.

Herb.

Departmental distribution: Central, Guairá.

Voucher: *F. Mereles 9938* (FCQ).

***Euphorbia serpens*** Kunth var. ***serpens***, Nov. Gen. Sp. 2: 52. 1817.

Syn.: *Chamaesyce serpens* (Kunth) Small

Herb.

Departmental distribution: Alto Paraguay, Amambay, Boquerón, Central, Presidente Hayes.

Voucher: *F. Mereles & S. Soria 9986* (BM, FCQ, G).

***Jatropha hieronymi*** Kuntze, Revis. Gen. Pl. 3(2): 287. 1898.

Shrub or small tree.

Departmental distribution: Alto Paraguay, Boquerón.

Voucher: *F. Mereles 10192* (FCQ).

#### FABACEAE

***Aeschynomene falcata*** (Poir.) DC. var. ***falcata***, Prodr. 2: 322. 1825.

Syn.: *Aeschynomene apoloana* Rusby, Aeschynomene falcata (Poir.) DC. var. paucijuga Benth., *Hedysarum diffusum* Vell., *Hedysarum falcatum* Poir.

Herb.

Departmental distribution: Caaguazú, Canindeyú, Central, Concepción, Cordillera, Guairá, Paraguarí.

Voucher: *F. Mereles 1883* (FCQ).

***Bauhinia forficata*** Link subsp. ***pruinosa*** (Vogel) Fortunato & Wunderlin, Darwiniana 27: 550. 1986.

Syn.: *Bauhinia candicans* Benth., *Bauhinia forficata* auct. non Link, *Bauhinia forficata* auct. non Hook. & Arn., Bauhinia forficata var. candicans (Benth.) Hassl. ex Latzina, Bauhinia forficata Link var. pruinosa (Vogel) Hassl., *Bauhinia pruinosa* Vogel, *Pauletia candicans* (Benth.) A.Schmitz, *Pauletia pruinosa* (Vogel) A.Schmitz

Shrub or tree.

Departmental distribution: Alto Paraná, Caaguazú, Central, Cordillera, Itapúa.

Voucher: *F. Mereles et al. 9905* (FCQ, G).

***Chamaecrista rotundifolia*** (Pers.) Greene var. ***rotundifolia***, Pittonia 4(20): 31. 1899.

Syn.: *Cassia bifoliolata* DC. ex Collad., Cassia bifoliolata var. pentandra (Raddi) Desv., Cassia bifoliolata var. rotundifolia (Pers.) Desv., comb. illeg., *Cassia fabaginifolia* Kunth, *Cassia monophylla* Vell., *Cassia pentandra* Raddi, *Cassia pentandria* Larrañaga, hom. illeg., *Cassia rotundifolia* Pers., *Cassia tenuivenosa* A.P.D.Jones., *Chamaecrista bifoliolata* (DC. ex Collad.) Greene.

Herb or subshrub.

Departmental distribution: Alto Paraguay, Amambay, Caaguazú, Caazapá, Central, Cordillera, Guairá.

Voucher: *J. De Egea et al. 1337* (BM, FCQ).

***Crotalaria incana*** L., Sp. Pl. 2: 716. 1753.

Syn.: *Chrysocalyx schimperi* Hochst. ex A.Rich., *Crotalaria affinis* DC., *Crotalaria criocaula* S.Schauer, *Crotalaria cubensis* DC., *Crotalaria diffusa* Vell., hom. illeg., *Crotalaria eriocaula* S.Schauer, *Crotalaria glabrescens* Andersson, hom. illeg., *Crotalaria herbacea* Schweig. ex Schrank, *Crotalaria hirta* Lag., hom. illeg., Crotalaria incana L. f. microphylla Chodat & Hassl., *Crotalaria megapotamica* Burkart, *Crotalaria montana* A.Rich., *Crotalaria picensis* Phil., *Crotalaria pubescens* Moench, *Crotalaria purpurascens* Lam., *Crotalaria radiata* Merr., *Crotalaria setifera* DC., *Lupinus rotundifolius* Sessé & Moç.

Herb or shrub.

Departmental distribution: Alto Paraguay, Amambay, Boquerón, Caaguazú, Caazapá, Canindeyú, Central, Concepción, Cordillera, Guairá, Itapúa, Paraguarí, Presidente Hayes, San Pedro.

Voucher: *J. De Egea et al. 1240* (FCQ).

***Desmodium cuneatum*** Hook. & Arn., Bot. Misc. 3: 195. 1832.

Syn.: *Desmodium brevipes* Vogel, *Meibomia brevipes* (Vogel) Kuntze, *Meibomia cuneata* (Hook. & Arn.) Kuntze

Subshrub.

Departmental distribution: Alto Paraguay, Amambay, Caazapá, Canindeyú, Central, Concepción, Cordillera, Guairá, Itapúa, Misiones, Ñeembucú, Paraguarí, Presidente Hayes, San Pedro.

Voucher: *J. De Egea et al. 1338* (BM, FCQ).

***Desmodium tortuosum*** (Sw.) DC., Prodr. 2: 332. 1825.

Syn.: *Desmodium purpureum* (Mill.) Fawc. & Rendle, hom. illeg., *Hedysarum purpureum* Mill., *Hedysarum tortuosum* Sw., *Meibomia purpurea* (Mill.) Vail, *Meibomia tortuosa* (Sw.) Kuntze.

Subshrub.

Departmental distribution: Amambay, Caaguazú, Concepción, Itapúa, Paraguarí.

Voucher: *F. Mereles & F. González Parini 7837* (FCQ).

***Indigofera bongardiana*** (Kuntze) Burkart var. ***bongardiana***, Darwiniana 4: 171. 1942.

Syn.: *Anila bongardiana* Kuntze, *Indigofera gracilis* Bong. ex Benth.

Herb.

Departmental distribution: Amambay, Caaguazú, Canindeyú, Central, Itapúa, Misiones, San Pedro.

Voucher: *F. Mereles & J. De Egea 10132* (FCQ, G).

***Lupinus gibertianus*** C.P.Sm. var. ***gibertianus***, Spec. Lupinorum 206. 1940.

Syn.: *Cytisus heptaphyllus* Vell., *Lupinus aspersus* C.P.Sm., *Lupinus bonplandianus* C.P.Sm., *Lupinus hassleranus* C.P.Sm., *Lupinus heptaphyllus* (Vell.) Hassl., Lupinus heptaphyllus f. hilarianus Benth., Lupinus heptaphyllus f. typicus Hassl., nom. inval., *Lupinus hilarianus* Benth., nom. superfl., *Lupinus propedubius* C.P.Sm., *Lupinus sanctae-anae* C.P.Sm., *Lupinus subumbellatus* C.P.Sm.

Herb.

Departmental distribution: Itapúa, Ñeembucú, San Pedro.

Voucher: *J. De Egea et al. 1283* (FCQ).

***Senna morongii*** (Britton) H.S.Irwin & Barneby, Mem. New York Bot. Gard. 35: 364. 1982.

Syn.: *Cassia acinacicarapa* Rusby, *Cassia cochabambae* Herzog, *Cassia morongii* Britton, *Cassia rojasiana* Hassl., Cassia tomentosa L. f. var. paucijuga Kuntze

Subshrub.

Departmental distribution: Alto Paraguay, Boquerón, Concepción, Misiones, Paraguarí, Presidente Hayes.

Voucher: *F. Mereles & S. Soria 9958* (BM, FCQ, G).

***Senna obtusifolia*** (L.) H.S.Irwin & Barneby, Mem. New York Bot. Gard. 35: 252. 1982.

Syn.: *Cassia humilis* Collad., *Cassia obtusifolia* L., Cassia tora L. var. humilis Collad., *Cassia toroides* Raf., *Cassia toroides* Roxb., nom. nud., *Diallobus falcatus* Raf., nom. illeg., *Diallobus uniflorus* Raf., *Emelista tora* (L.) Britton & Rose, *Senna toroides* Roxb.

Herb or subshrub.

Departmental distribution: Alto Paraguay, Alto Paraná, Boquerón, Caaguazú, Caazapá, Canindeyú, Central, Cordillera, Guairá, Itapúa, Paraguarí, Presidente Hayes.

Voucher: *J. De Egea et al. 1312* (FCQ).

***Senna occidentalis*** (L.) Link., Handbuch 2: 140. 1831.

Syn.: *Cassia caroliniana* Walter, *Cassia ciliata* Raf., *Cassia falcata* L., *Cassia foetida* Pers., nom. illeg., *Cassia macradena* Collad., *Cassia obliquifolia* Schrank, *Cassia occidentalis* L., Cassia occidentalis var. aristata Collad., *Cassia planisiliqua* L., *Cassia planisiliqua* Lam., hom. illeg., *Cassia plumieri* DC., *Ditremexa occidentalis* (L.) Britton & Rose ex Britton & Wilson, *Senna occidentalis* (L.) Roxb., comb. superfl.

Herb or subshrub.

Departmental distribution: Alto Paraguay, Amambay, Boquerón, Caaguazú, Caazapá, Canindeyú, Central, Concepción, Cordillera, Guairá, Misiones, Ñeembucú, Paraguarí, Presidente Hayes, San Pedro.

Voucher: *J. De Egea et al. 1262* (FCQ).

***Vicia epetiolaris*** Burkart var. ***epetiolaris***, Darwiniana 14: 182. 1966.

Vine.

Departmental distribution: Central, Misiones, Presidente Hayes.

Voucher: *G. Céspedes 439* (FCQ).

#### LAMIACEAE

***Hyptis lappacea*** Benth., Labiat. Gen. Sp. 103. 1833.

Syn.: *Hyptis cinerea* Morong, Hyptis cinerea var. genuina Briq., nom. inval., Hyptis cinerea var. stenophylla Briq., *Hyptis globifera* auct. non G.Mey., *Hyptis michelii* Briq. ex Micheli, *Hyptis trichoneura* Briq. ex Micheli, *Mesosphaerum cinereum* (Morong) Briq., *Mesosphaerum lappaceum* (Benth.) Kuntze, *Mesosphaerum trichoneurum* (Briq. ex Micheli) Briq.

Herb.

Departmental distribution: Alto Paraguay, Alto Paraná, Amambay, Boquerón, Caaguazú, Caazapá, Central, Concepción, Cordillera, Guairá, Ñeembucú, Paraguarí, Presidente Hayes.

Voucher: *R. Degen 1434* (FCQ).

***Leonotis nepetifolia*** (L.) R.Br., Hortus Kew. (2nd ed.) 3: 409. 1811.

Syn.: *Phlomis nepetaefolia L*.

Herb. Introduced.

Departmental distribution: Amambay, Caazapá, Central, Cordillera, Paraguarí, San Pedro.

Voucher: *E. Zardini 8223* (FCQ, MO).

►***Leonurus cardiaca*** L., Sp. Pl. 2: 584. 1753.

Herb. Introduced.

Departmental distribution: Guairá.

Voucher: *R. Degen et al. 3811* (FCQ).

***Leonurus japonicus*** Houtt., Nat. Hist. 2(9): 366, t. 57, f. 1. 1778.

Syn.: *Leonurus sibiricus* auct. non L.

Herb. Introduced.

Departmental distribution: Alto Paraná, Caazapá, Central, Guairá, Itapúa, Paraguarí.

Voucher: *R. Degen 1516* (FCQ).

***Scutellaria racemosa*** Pers., Syn. Pl. 2(1): 136. 1806.

Syn.: *Scutellaria bonariensis* Willd. ex Benth., *Scutellaria hastata* Larrañaga, *Scutellaria heterophylla* Willd. ex Benth., *Scutellaria rumicifolia* Kunth, *Scutellaria rojasii* Briq.

Herb.

Departmental distribution: Alto Paraguay, Caaguazú, Canindeyú, Central, Concepción, Cordillera, Itapúa, Misiones, Ñeembucú, Paraguarí, Presidente Hayes, San Pedro.

Voucher: *J. De Egea 1299* (BM, FCQ).

#### LYTHRACEAE

***Cuphea carthagenensis*** (Jacq.) J.F.Macbr., Publ. Field Mus. Nat. Hist., Bot. Ser. 8: 124. 1930.

Syn.: *Cuphea balsamona* Cham. & Schltdl., *Lythrum carthagenense* Jacq.

Herb or subshrub.

Departmental distribution: Amambay, Caazapá, Canindeyú, Central, Concepción, Cordillera, Guairá, Itapúa, Misiones, Ñeembucú, Paraguarí, Presidente Hayes, San Pedro.

Voucher: *N. Soria 3111a* (FCQ).

***Cuphea racemosa*** (L.f.) Spreng. subsp. ***racemosa***, Syst. Veg. (ed. 16) 2: 455. 1825.

Syn.: *Cuphea obtusifolia* Koehne ex Bacig., *Cuphea origanifolia* Cham. & Schltdl., Cuphea racemosa var. discolor Lourteig, *Lythrum racemosum* L.f.

Herb or subshrub.

Departmental distribution: Alto Paraná, Amambay, Caaguazú, Caazapá, Canindeyú, Central, Concepción, Cordillera, Guairá, Itapúa, Misiones, Ñeembucú, Paraguarí, Presidente Hayes, San Pedro.

Voucher: *G. Céspedes 428* (FCQ).

***Heimia salicifolia*** Link., Icon. Pl. Select. 63, t. 28. 1822.

Syn.: *Nesaea salicifolia* Kunth

Shrub or subshrub.

Departmental distribution: Alto Paraguay, Alto Paraná, Amambay, Boquerón, Caaguazú, Caazapá, Canindeyú, Central, Concepción, Cordillera, Guairá, Itapúa, Misiones, Ñeembucú, Paraguarí, Presidente Hayes, San Pedro.

Voucher: *F. Mereles et al. 10012* (BM, FCQ, G).

***Pleurophora saccocarpa*** Koehne, Bot. Jahrb. Syst. 2(5): 426. 1882.

Syn.: *Pleurophora annulosa* Koehne, Pleurophora saccocarpa Koehne var. fiebrigii Koehne, Pleurophora saccocarpa var. glabrescens Koehne, Pleurophora saccocarpa var. hirtella Koehne, Pleurophora saccocarpa var. velutina Koehne

Shrub.

Departmental distribution: Alto Paraguay, Alto Paraná, Amambay, Boquerón, Canindeyú, Central, Concepción, Misiones, Ñeembucú, Paraguarí, Presidente Hayes, San Pedro.

Voucher: *F. Mereles & J. De Egea 10137* (BM, FCQ, G).

#### MALVACEAE

***Abutilon pauciflorum*** A.St.-Hil., Fl. Bras. Merid. 1: 206. 1827.

Syn.: *Abutilon melanocarpum* A.St.-Hil. & Naudin, *Abutilon mollissimum* auct. non (Cav.) Sweet, Abutilon pauciflorum A.St.-Hil. var. cano-tomentosum Hassl., Abutilon pauciflorum f. longe-corniculatum Hassl., *Abutilon pedunculare* auct. non Humb., Bonpl. & Kunth, *Abutilon rugosulum* Hochr. ex Chodat & Hassl.

Shrub.

Departmental distribution: Central, Concepción, Cordillera, Guairá, Ñeembucú, Paraguarí, Presidente Hayes.

Voucher: *I. Basualdo 17* (FCQ).

***Corchorus hirtus*** L., Sp. Pl., ed. 2. 2: 747. 1762.

Syn.: Corchorus hirtus L. var. cuyabensis K.Schum, Corchorus hirtus var. orinocensis (Kunth) K.Schum., *Corchorus orinocensis* Kunth, *Corchorus pilolobus* Link

Herb or subshrub.

Departmental distribution: Alto Paraguay, Alto Paraná, Amambay, Boquerón, Canindeyú, Central, Concepción, Cordillera, Ñeembucú, Paraguarí, Presidente Hayes, San Pedro.

Voucher: *M. Dematteis et al. 2927* (FCQ).

***Malvastrum coromandelianum*** (L.) Gracke subsp. ***coromandelianum***, Bonplandia 5(18): 295. 1857.

Syn.: *Malva coromandeliana* L., *Malva tricuspidata* Aiton, *Malvastrum carpinifolium* A.Gray, Malvastrum coromandelianum (L.) Garcke f. breviaristata Hassl., *Malvastrum tricuspidatum* (Aiton) A.Gray, *Malveopsis coromandeliana* (L.) Morong

Subshrub.

Departmental distribution: Alto Paraguay, Amambay, Boquerón, Caaguazú, Central, Concepción, Cordillera, Guairá, Itapúa, Paraguarí, Presidente Hayes, San Pedro.

Voucher: *F. Mereles et al. 9926* (FCQ).

***Melochia canescens*** Cristóbal, Bonplandia 9(1–2): 43. 1996.

Subshrub.

Departmental distribution: Alto Paraguay, Boquerón, Ñeembucú, Presidente Hayes.

Voucher: *F. Mereles & S. Soria 9947* (BM, CTES, FCQ, G).

***Melochia hermannioides*** A.St.-Hil., Fl. Bras. Merid. 1(4): 163. 1825.

Syn.: Melochia hermannioides f. heterophylla Hassl., Melochia hermannioides var. lacinulata (K.Schum. & Hassl.) Hassl., Melochia hermannioides var. lanceolata Hassl., Melochia hermannioides f. typica Hassl., nom. inval., *Melochia lacinulata* K.Schum. & Hassl., Melochia parvifolia Kunth f. roseiflora K.Schum. & Hassl.

Herb or subshrub.

Departmental distribution: Caaguazú, Canindeyú, Central, Concepción, Cordillera, Guairá, Misiones, Ñeembucú, Paraguarí, Presidente Hayes, San Pedro.

Voucher: *F. Mereles & J. De Egea 10141* (FCQ, G).

***Melochia ministella*** Cristóbal, Bonplandia 9(1–2): 46. 1996.

Subshrub.

Departmental distribution: Alto Paraguay, Concepción, Itapúa, Presidente Hayes.

Voucher: *F. Mereles et al. 9925* (BM, FCQ, G).

***Melochia pyramidata*** L. var. ***hieronymi*** K.Schum., Fl. Bras. 12(3): 35. 1886.

Syn.: Melochia pyramidata L. f. intermedia Hassl., Melochia pyramidata var. pseudotomentosa Hassl., Melochia pyramidata f. transitoria K.Schum. & Hassl., Melochia tomentosa L. var. mattogrossensis R.E.Fr.

Subshrub.

Departmental distribution: Alto Paraguay, Amambay, Caaguazú, Caazapá, Canindeyú, Central, Concepción, Cordillera, Guairá, Itapúa, Misiones, Ñeembucú, Paraguarí, Presidente Hayes, San Pedro.

Voucher: *C.L. Cristóbal & A. Krapovickas 2293* (FCQ).

***Melochia pyramidata*** L. var. ***pyramidata***, Sp. Pl. 2: 674. 1753.

Subshrub.

Departmental distribution: Alto Paraguay, Amambay, Boquerón, Central, Concepción, Itapúa, Misiones, Paraguarí, Presidente Hayes, San Pedro.

Voucher: *R. Spichiger et al. RS2621* (FCQ).

***Pavonia sidifolia*** Kunth, Nov. Gen. Sp. 5: 283. 1822.

Syn.: *Asterochlaena sidifolia* (Kunth) Hassl., Asterochlaena sidifolia ssp. diuretica (A.St.-Hil.) Hassl., Asterochlaena sidifolia var. paraguayensis Hassl., *Pavonia diuretica* A.St.-Hil.

Shrub or subshrub.

Departmental distribution: Alto Paraguay, Amambay, Canindeyú, Central, Concepción, Cordillera, Guairá, Misiones, Paraguarí, Presidente Hayes, San Pedro.

Voucher: *A. Schinini 23872* (FCQ).

***Pseudabutilon callimorphum*** (Hochr.) R.E.Fr. var. ***callimorphum***, Kungl. Svenska Vetenskapsakad. Handl. 43(4): 105. 1908.

Syn.: *Sida callimorpha* Hochr., *Wissadula callimorpha* (Hochr.) Hassl.

Subshrub.

Departmental distribution: Alto Paraguay, Boquerón, Concepción, Presidente Hayes.

Voucher: *F. Mereles & S. Soria 9939* (BM, CTES, FCQ, G).

***Sida cordifolia*** L., Sp. Pl. 2: 684. 1753.

Syn.: *Malvastrum cordifolium* Rojas Acosta, *Sida altheifolia* Sw., Sida cordifolia L. var. breviaristata Monteiro, *Sida rotundifolia* Lam.

Subshrub.

Departmental distribution: Alto Paraguay, Amambay, Boquerón, Caaguazú, Caazapá, Central, Concepción, Cordillera, Guairá, Paraguarí, Presidente Hayes, San Pedro.

Voucher: *J. De Egea et al. 1264* (FCQ).

***Sida linifolia*** Cav., Diss. 1, Diss. Bot. 14 (t. 2, f. 1). 1785.

Syn.: *Sida fiebrigii* Ulbr., Sida linifolia Juss. ex Cav. f. flaviflora Chodat & Hassl.

Herb.

Departmental distribution: Amambay, Caaguazú, Central, Concepción, Cordillera, Guairá, Paraguarí, San Pedro.

Voucher: *F. Mereles 3983* (FCQ).

***Sida rhombifolia*** L., Sp. Pl. 2: 684. 1753.

Syn.: Sida rhombifolia L. var. canariensis (Willd.) Griseb., Sida rhombifolia var. rhomboidea (Roxb.) Mast., *Sida rhomboidea* Roxb., *Sida subrhombiformis* Larrañaga

Herb or subshrub.

Departmental distribution: Alto Paraguay, Amambay, Boquerón, Caaguazú, Central, Cordillera, Guairá, Itapúa, Ñeembucú, Paraguarí, Presidente Hayes, San Pedro.

Voucher: *J. De Egea et al. 1231* (FCQ).

***Sida santaremensis*** Monteiro, Monogr. Malv. Brasil. Fasc. I. 44. 1936.

Syn.: *Sida glaziovii* auct. non K.Schum., Sida rhombifolia L. var. subtomentosa K.Schum., Sida santaremensis Monteiro var. krapovickasiana Monteiro

Subshrub.

Departmental distribution: Alto Paraguay, Amambay, Boquerón, Caaguazú, Central, Concepción, Cordillera, Guairá, Paraguarí, Presidente Hayes, San Pedro.

Voucher: *N. Soria 1234* (FCQ).

***Sida spinosa*** L., Sp. Pl. 2: 683. 1753.

Syn.: *Sida angustifolia* Lam., *Sida escobilla* Larrañaga, Sida spinosa L. var. angustifolia (Lam.) Griseb.

Subshrub.

Departmental distribution: Alto Paraguay, Boquerón, Central, Cordillera, Guairá, Paraguarí, Presidente Hayes.

Voucher: *E. Bordas 4198* (FCQ).

***Sidastrum paniculatum*** (L.) Fryxell, Brittonia 30(4): 453. 1978.

Syn.: *Sida paniculata* L.

Subshrub.

Departmental distribution: Alto Paraguay, Caaguazú, Canindeyú, Central, Concepción, Cordillera, Guairá, Misiones, Ñeembucú, Paraguarí, Presidente Hayes, San Pedro.

Voucher: *G. Schmeda 314* (FCQ).

***Triumfetta semitriloba*** Jacq., Enum. Syst. Pl. 22. 1760.

Syn.: *Triumfetta abutiloides* A.St.-Hil., *Triumfetta tricuspis* A.St.-Hil.

Shrub.

Departmental distribution: Alto Paraná, Amambay, Caaguazú, Caazapá, Canindeyú, Central, Concepción, Cordillera, Guairá, Itapúa, Misiones, Ñeembucú, Paraguarí, San Pedro.

Voucher: *I. Basualdo 818* (FCQ).

***Waltheria indica*** L., Sp. Pl. 2: 673. 1753.

Syn.: *Waltheria americana* L., Waltheria americana var. indica (L.) K.Schum.

Subshrub.

Departmental distribution: Alto Paraguay, Alto Paraná, Amambay, Boquerón, Caaguazú, Canindeyú, Central, Concepción, Cordillera, Guairá, Itapúa, Misiones, Paraguarí, Presidente Hayes, San Pedro.

Voucher: *I. Basualdo 179* (FCQ).

***Wissadula densiflora*** R.E.Fr. var. ***densiflora***, Kungl. Svenska Vetenskapsakad. Handl. 43(4): 64, pl. 4, 6. 1908.

Subshrub.

Departmental distribution: Alto Paraguay, Boquerón, Presidente Hayes.

Voucher: *F. Mereles & S. Soria 9948* (BM, FCQ, G).

***Wissadula subpeltata*** (Kuntze) R.E.Fr., Kungl. Svenska Vetenskapsakad. Handl. 43(4): 56, pl. 5, 6, 7. 1908.

Syn.: Abutilon amplissimum (L.) Kuntze var. subpeltatum Kuntze, *Wissadula amplissima* auct. non (L.) R.E.Fr.

Shrub.

Departmental distribution: Alto Paraguay, Alto Paraná, Amambay, Caaguazú, Concepción, Cordillera, Guairá, Paraguarí, Presidente Hayes.

Voucher: *M. Dematteis et al. 2788* (FCQ).

#### MOLLUGINACEAE

***Mollugo verticillata*** L., Sp. Pl. 1: 89. 1753.

Syn.: *Mollugo arenaria* Kunth

Herb.

Departmental distribution: Alto Paraguay, Amambay, Boquerón, Caazapá, Central, Concepción, Cordillera, Paraguarí, Presidente Hayes.

Voucher: *G. Céspedes 424* (FCQ).

#### MORACEAE

***Dorstenia brasiliensis*** Lam., Encycl. 2(1): 317. 1786.

Syn.: Dorstenia brasiliensis Lam. f. balansae Chodat, Dorstenia brasiliensis var. guaranitica Chodat, hom. illeg., *Dorstenia brasiliensis* var. major Chodat & Hassl., Dorstenia brasiliensis var. palustris Hassl., Dorstenia brasiliensis var. tomentosa (Fisch. & C.A.Mey.) Hassl., Dorstenia brasiliensis var. tubicina (Ruiz & Pav.) Chodat & Vischer Dorstenia brasiliensis var. typica Hassl., nom. inval., *Dorstenia montevidensis* Field & Gardner, *Dorstenia schulzii* Carauta, C.Valente & Dunn de Araujo, *Dorstenia tomentosa* Fisch. & C.A.Mey., *Dorstenia tubicina* Ruiz & Pav., Dorstenia tubicina var. genuina Hassl., nom. inval., Dorstenia tubicina f. major Hassl., Dorstenia tubicina var. opifera (Mart.) Hassl. Dorstenia tubicina f. subexcentrica Hassl., Dorstenia tubicina f. typica Hassl., nom. inval.

Herb.

Departmental distribution: Alto Paraguay, Amambay, Caaguazú, Central, Cordillera, Guairá, Ñeembucú, Presidente Hayes, San Pedro.

Voucher: *J. De Egea et al. 1255* (FCQ).

#### NYCTAGINACEAE

***Boerhavia diffusa*** L. var. ***diffusa***, Sp. Pl. 1: 3. 1753.

Syn.: Boerhavia coccinea Mill. var. paniculata (Rich.) Moscoso, *Boerhavia paniculata* Rich.

Herb. Introduced.

Departmental distribution: Alto Paraguay, Boquerón, Caazapá, Central, Guairá, Presidente Hayes.

Voucher: *F. Mereles & S. Soria 9985* (BM, FCQ, G).

***Boerhavia diffusa*** L. var. ***leiocarpha*** (Heimer) C.D.Adams, Mitt. Bot. Staatssamml. München 8: 115. 1970.

Syn.: *Boerhavia ciliato-bracteata* Heimerl, Boerhavia coccinea Mill. var. leiocarpa (Heimerl) Standl., Boerhavia diffusa L. var. paniculata D.Parodi, nom. nud., *Boerhavia friesii* Heimerl, Boerhavia paniculata Rich. var. guaranitica Heimerl, Boerhavia paniculata var. leiocarpa Heimerl, Boerhavia paniculata f. leiocarpa Heimerl

Herb. Introduced.

Departmental distribution: Alto Paraguay, Cordillera, Presidente Hayes.

Voucher: *F. Mereles & S. Soria 10184* (FCQ).

#### ONAGRACEAE

***Ludwigia lagunae*** (Morong) H.Hara, J. Jap. Bot. 28: 292. 1953.

Syn.: Jussiaea brachycarpa Lam. ssp. epilobioides (Chodat & Hassl.) Hassl., Jussiaea brachycarpa var. genuina Hassl., nom. inval., Jussiaea brachycarpa var. grandiflora Hassl., Jussiaea brachycarpa var. paraguayensis (Chodat) Hassl., Jussiaea brachycarpa var. parviflora (Chodat & Hassl.) Hassl., Jussiaea brachycarpa var. puberula Hassl., *Jussiaea brachycarpa* Micheli, hom. illeg., *Jussiaea epilobioides* Chodat & Hassl., Jussiaea epilobioides var. parviflora Chodat & Hassl., *Jussiaea lagunae* Morong, Jussiaea lagunae var. paraguayensis (Chodat) Munz, Jussiaea lagunae var. typica Munz, nom. inval., *Jussiaea leveilleana* Bertoni, *Jussiaea paraguayensis* Chodat, Jussiaea suffruticosa L. var. epilobioides (Chodat & Hassl.) Bertoni, Jussiaea suffruticosa var. paraguayensis (Chodat) Bertoni, Jussiaea suffruticosa var. parviflora (Chodat & Hassl.) Bertoni

Herb or subshrub.

Departmental distribution: Alto Paraguay, Amambay, Caaguazú, Canindeyú, Central, Concepción, Cordillera, Guairá, Misiones, Ñeembucú, Paraguarí, Presidente Hayes, San Pedro.

Voucher: *F. Mereles & J. De Egea 10151* (FCQ, G).

#### OXALIDACEAE

***Oxalis conorrhiza*** Jacq., Monographia, Iconibus Illustrata 26. 1794.

Syn.: *Acetosella caespitosa* (A.St.-Hil.) Kuntze, *Acetosella chrysantha* (Progel) Kuntze, *Acetosella cineracea* (A.St.-Hil.) Kuntze, *Acetosella commersonii* (Pers.) Kuntze, *Acetosella conorrhiza* (Jacq.) Kuntze, *Acetosella megapotamica* (Spreng.) Kuntze, *Oxalis andicola* Gillies ex Hook. & Arn., Oxalis andicola var. wallichiana Gillies ex Hook. & Arn., *Oxalis brevipes* Fredr., *Oxalis caespitosa* A.St.-Hil., *Oxalis chrysantha* Progel, Oxalis chrysantha var. pusilla Progel, *Oxalis cineracea* A.St.-Hil., *Oxalis commersonii* Pers., *Oxalis conorhixa* Larrañaga, *Oxalis cordobensis* R.Knuth, Oxalis cordobensis var. humilior R.Knuth, Oxalis corniculata L. var. serpens R.Knuth, *Oxalis hassleriana* Chodat, *Oxalis linneaformis* R. Knuth, *Oxalis megapotamica* Spreng., Oxalis repens Thunb. f. uniflora Hieron. & Lorentz ex R.Knuth, nom. nud., *Oxalis sexenata* Savigny, *Oxalis sternbergii* auct. non Zucc., *Xanthoxalis chrysantha* (Progel) Holub, *Xanthoxalis commersonii* (Pers.) Holub, *Xanthoxalis cordobensis* (R.Knuth) Holub

Herb.

Departmental distribution: Alto Paraná, Alto Paraguay, Amambay, Caaguazú, Central, Concepción, Cordillera, Misiones, Presidente Hayes.

Voucher: *F. Mereles et al. 8100* (FCQ).

***Oxalis corniculata*** L. var. ***corniculata***., Sp. Pl. 1: 435. 1753.

Syn.: *Acetosella corniculata* (L.) Kuntze, Acetosella corniculata var. repens (Thunb.) Kuntze, *Oxalis steudeliana* R.Knuth, *Xanthoxalis corniculata* (L.) Small

Herb.

Departmental distribution: Alto Paraná, Central, Cordillera, Guairá.

Voucher: *E. Zardini 23445* (AS, MO).

***Oxalis paludosa*** A.St.-Hil., Fl. Bras. Merid. 1: 121. 1825.

Syn.: *Acetosella montevidensis* (Progel) Kuntze, *Acetosella paludosa* (A.St.-Hil.) Kuntze, *Oxalis corrientesensis* R.Knuth, *Oxalis duricaulis* R.Knuth, *Oxalis montevidensis* Progel

Herb.

Departmental distribution: Central, Cordillera, Ñeembucú, Paraguarí, Presidente Hayes.

Voucher: *G. Céspedes 438* (FCQ).

#### PASSIFLORACEAE

***Turnera grandidentata*** (Urb.) Arbo, Candollea 40(1): 176. 1985.

Syn.: Turnera ulmifolia L. var. elegans auct. non (Otto) Urb.,Turnera ulmifolia L. var. grandidentata Urb.

Herb. Endemic.

Departmental distribution: Caaguazú, Central, Concepción, Cordillera, Misiones, Paraguarí, San Pedro.

Voucher: *F. González & M. J. López 554* (FCQ).

#### PHYLLANTHACEAE

***Phyllanthus niruri*** L., Sp. Pl. 2: 981. 1753.

Syn.: *Phyllanthus lathyroides* Kunth, Phyllanthus lathyroides f. rosellus Müll.Arg., Phyllanthus niruri L. ssp. lathyroides (Kunth) G.L.Webster, *Phyllanthus microphyllus* Mart., Phyllanthus niruri f. microphyllus (Müll.Arg.) G.L.Webster, *Phyllanthus rosellus* (Müll.Arg.) Müll.Arg.

Herb.

Departmental distribution: Caazapá, Canindeyú, Central, Concepción, Cordillera, Guairá, Itapúa, Ñeembucú, Paraguarí.

Voucher: *E. Zardini et al. 2421* (FCQ, MO).

***Phyllanthus orbiculatus*** Rich., Actes Soc. Hist. Nat. Paris 1:113. 1792.

Syn.: *Orbicularia orbiculata* (Rich.) Moldenke

Herb.

Departmental distribution: Alto Paraná, Amambay, Canindeyú, Central, Concepción, Cordillera, Guairá, Paraguarí.

Voucher: *E. Zardini & R. Degen 3767* (FCQ, MO).

#### PLANTAGINACEAE

***Plantago tomentosa*** Lam. ssp. ***tomentosa***, Tabl. Encycl. 1(2): 340. 1792.

Syn.: *Plantago achalensis* Pilg., Plantago achalensis var. hirtula Pilg., Plantago achalensis f. minor Pilg., *Plantago affinis* Decne., *Plantago arechavaletai* Pilg., Plantago bicallosa Decne. var. hirsutior Pilg., *Plantago grisebachii* Hieron., *Plantago hypolasia* Pilg., *Plantago hypoleuca* Pilg., Plantago oreades Decne. var. lanuginosa Griseb, *Plantago paralias* Decne., Plantago paralias var. achalensis (Pilg.) Pilg., Plantago paralias ssp. affinis (Decne.) Pilg., Plantago paralias var. brevifolia Pilg., Plantago paralias var. cordobensis (Pilg.) Pilg., Plantago paralias ssp. dasystachys (Pilg.) Pilg., Plantago paralias ssp. euparalias Pilg., nom. inval., Plantago paralias var. glabrescens (Pilg.) Pilg, Plantago paralias ssp. grisebachii (Hieron.) Pilg., Plantago paralias ssp. hypolasia (Pilg.) Pilg., Plantago paralias var. lasiophylla (Pilg.) Pilg, Plantago paralias ssp. leiocalyx (Pilg.) Pilg., Plantago paralias var. mollior (Pilg.) Pilg, Plantago paralias ssp. petiolata (Pilg.) Pilg., Plantago paralias var. saxicola (Pilg.) Pilg, Plantago paralias ssp. schlechtendaliana (Pilg.) Pilg., Plantago paralias ssp. selloana (Pilg.) Pilg., Plantago tomentosa var. achalensis Pilg., Plantago tomentosa ssp. balansai Pilg., Plantago tomentosa var. brevifolia Pilg., Plantago tomentosa var. cordobensis Pilg., Plantago tomentosa ssp. dasystachys Pilg., Plantago tomentosa var. glabrescens Schmidt, Plantago tomentosa Lam. ssp. grisebachii (Hieron.) Pilg., Plantago tomentosa ssp. hypolasia (Pilg.) Pilg., Plantago tomentosa var. lasiophylla Pilg., Plantago tomentosa ssp. leiocalyx Pilg., Plantago tomentosa var. mollior Pilg., Plantago tomentosa ssp. paralias (Decne.) Pilg., Plantago tomentosa ssp. petiolata Pilg., Plantago tomentosa var. saxicola Pilg, Plantago tomentosa ssp. schlechtendaliana Pilg., Plantago tomentosa ssp. selloana Pilg.

Herb.

Departmental distribution: Alto Paraguay, Caaguazú, Caazapá, Canindeyú, Central, Cordillera, Guairá, Ñeembucú, Paraguarí, Presidente Hayes, SanPedro

Voucher: *R. Degen et al. 4348* (FCQ).

***Scoparia dulcis*** L., Sp. Pl. 1: 116. 1753.

Syn.: *Capraria dulcis* (L.) Kuntze, Scoparia dulcis L. var. tenuifolia Griseb., *Scoparia nudicaulis* Chodat & Hassl., *Scoparia procumbens* Jacq., *Scoparia purpurea* Ridl.

Herb or subshrub.

Departmental distribution: Alto Paraguay, Alto Paraná, Amambay, Caaguazú, Caazapá, Canindeyú, Central, Concepción, Cordillera, Guairá, Misiones, Ñeembucú, Paraguarí, Presidente Hayes, San Pedro.

Voucher: *F. Mereles et al. 10072* (FCQ).

***Scoparia ericacea*** Cham. & Schltdl., Linnaea 2: 604. 1827.

Syn.: *Capraria ericacea* (Cham. & Schltdl.) Kuntze, *Scoparia divaricata* R.E.Fr., *Scoparia plebeja* Cham. & Schltdl.

Herb or subshrub.

Departmental distribution: Alto Paraná, Cordillera, Presidente Hayes, San Pedro.

Voucher: *F. Mereles et al. 10071* (FCQ).

***Stemodia verticillata*** (Mill.) Hassl., Trab. Mus. Farmacol. 21: 110. 1909.

Syn.: *Capraria humilis* Sol., *Erinus verticillatus* Mill., *Lendneria humilis* (Sol.) Minod, *Lendneria verticillata* (Mill.) Britton, *Stemodia arenaria* Kunth, *Stemodia humilis* (Sol.) G.Dawson, *Stemodia macrotricha* Colla, *Stemodia parviflora* W.T.Aiton, *Stemodiacra verticillata* (Mill.) Kuntze

Herb.

Departmental distribution: Caazapá, Canindeyú, Cordillera, Guairá, Ñeembucú, San Pedro.

Voucher: *J. De Egea et al. 1220* (BM, FCQ).

#### POACEAE

***Andropogon bicornis*** L., Sp. Pl. 2: 1046. 1753.

Syn.: Andropogon bicornis L. var. absconditus Hack., Andropogon bicornis var. burchellii Hack., Andropogon bicornis var. gracillimus Hack., Andropogon bicornis var. virginicoides Hack.

Herb.

Departmental distribution: Alto Paraná, Amambay, Canindeyú, Central, Guairá, Itapúa, Misiones, Ñeembucú, Paraguarí, Presidente Hayes, San Pedro.

Voucher: *F. Mereles et al. 10179* (FCQ, G).

►***Avena sativa*** L. subsp. ***sativa***, Sp.Pl. 1: 79. 1753.

Syn.: Avena fatua L. f. glaberrima Thell., Avena fatua ssp. sativa (L.) Thell., Avena sativa L. var. glaberrima (Thell.) Parodi

Herb. Introduced.

Departmental distribution: Alto Paraná.

Voucher: *F. Mereles et al. 10074* (FCQ).

***Cenchrus ciliaris*** L., Mant. Pl. 2: 302. 1771.

Syn.: *Pennisetum cenchroides* Rich. ex Pers., nom. illeg., *Pennisetum ciliare* (L.) Link

Herb. Introduced.

Departmental distribution: Alto Paraguay, Boquerón, Guairá, Presidente Hayes.

Voucher: *F. Mereles & S. Soria 9969* (FCQ, G).

***Cenchrus echinatus*** L., Sp. Pl. 2: 1050. 1753.

Syn.: *Cenchrus crinitus* Mez, *Cenchrus echinatus* Cav., nom. inval.

Herb.

Departmental distribution: Alto Paraguay, Alto Paraná, Central, Concepción, Cordillera, Guairá, Itapúa, Paraguarí, Presidente Hayes, San Pedro.

Voucher: *J. De Egea et al. 1293* (FCQ).

***Cenchrus purpureus*** (Schumach.) Morrone, Ann. Bot. (Oxford) 106(1): 129. 2010.

Syn.: *Pennisetum purpureum* Schumach.

Herb. Intruduced.

Departmental distribution: Alto Paraná, Canindeyú, Central, Paraguarí.

Voucher: *F. Mereles et al. 10083* (BM, FCQ, G).

***Chloris virgata*** Sw., Fl. Ind. Occid. 1: 203. 1797.

Syn.: *Chloris compressa* DC., *Chloris elegans* Kunth

Herb.

Departmental distribution: Alto Paraguay, Boquerón.

Voucher: *F. Mereles & S. Soria 10195* (FCQ).

***Cynodon dactylon*** (L.) Pers. var. ***dactylon***, Syn. Pl. 1: 85. 1805.

Syn.: *Capriola dactylon* (L.) Kuntze, *Cynodon aristiglumis* Caro & E.A.Sánchez, *Cynodon aristulatus* Caro & E.A.Sánchez, *Cynodon erectus* J.Presl., *Cynodon pascurus* Nees, *Panicum dactyon* L.

Herb. Introduced.

Departmental distribution: Alto Paraná, Cordillera, Guairá, Itapúa, San Pedro.

Voucher: *J. De Egea et al. 1304* (FCQ).

***Cynodon dactylon*** (L.) Pers. var. ***longiglumis*** Caro & E.A.Sánchez, Kurtziana 5: 210, f. 2G. 1969.

Herb.

Departmental distribution: Itapúa, San Pedro.

Voucher: *J. De Egea et al. 1282* (FCQ).

***Digitaria aequiglumis*** (Hack. & Arechav.) Parodi var. ***aequiglumis***, Revista Fac. Agron. Veterin. 4: 47. 1922.

Syn.: *Digitaria campestris* Henrard, *Digitaria chillanensis* Phil. ex Henrard, nom. nud., *Panicum aequiglume* Hack. & Arechav., *Panicum debile* Phil., nom. illeg., Panicum debile Desf. var. aequiglume (Hack. & Arechav.) Hack., *Panicum ramosum* Arechav., *Panicum tridactylum* Phil., hom. illeg., *Syntherisma aequiglumis* (Hack. & Arechav.) Hitchc.

Herb.

Departmental distribution: Alto Paraná, Central, Itapúa, Presidente Hayes.

Voucher: *C.V.M. Pavetti 10974* (Zuloaga et al. 1994).

***Digitaria balansae*** Henrard, Meded. Rijks-Herb. 61: 2. 1930.

Herb.

Departmental distribution: Alto Paraná, Caaguazú, Caazapá, Cordillera, Guairá, Itapúa, Misiones, Paraguarí, Presidente Hayes.

Voucher: *T. Rojas 11188* (AS, MO).

***Digitaria bicornis*** (Lam.) Roem. & Schult., Syst. Veg. (ed. 15 bis) 2: 470. 1817.

Syn.: *Paspalum bicorne* Lam., *Panicum bicorne* (Lam.) Kunth, *Panicum glaucescens* Nees, hom. illeg.

Herb. Introduced.

Departmental distribution: Alto Paraguay, Alto Paraná, Amambay, Boquerón, Central, Concepción, Cordillera, Itapúa, Misiones, Presidente Hayes, San Pedro.

Voucher: *F. Mereles et al. 9918* (FCQ).

***Digitaria ciliaris*** (Retz.) Koeler, Descr. Gram. 27. 1802.

Syn.: *Digitaria abortiva* Reeder, *Digitaria adscendens* (Kunth) Henrard, *Digitaria marginata* Link, *Digitaria tarapacana* Phil., *Panicum adscendes* Kunth, *Panicum ciliare* Retz., Panicum sanguinale L. var. ciliare (Retz.) Vasey

Herb.

Departmental distribution: Alto Paraguay, Alto Paraná, Amambay, Caaguazú, Canindeyú, Central, Concepción, Cordillera, Guairá, Itapúa, Misiones, Paraguarí, Presidente Hayes, San Pedro.

Voucher: *F. Mereles et al. 10069* (FCQ).

***Digitaria horizontalis*** Willd., Enum. Pl. 92. 1809.

Syn.: *Panicum horizontale* (Willd.) G.Mey., *Panicum oxyanthum* Steud., Panicum sanguinale L. var. horizontale (Willd.) Schweinf.

Herb.

Departmental distribution: Alto Paraguay, Amambay, Boquerón, Canindeyú, Central, Concepción, Cordillera, Guairá, Itapúa, Misiones, Presidente Hayes, San Pedro.

Voucher: *W.J. Hahn 2076* (MO, PY).

***Digitaria insularis*** (L.) Fedde, Bot. Jahresber. 31(1): 778. 1904.

Syn.: *Acicarpa sacchariflora* Raddi, nom. illeg., *Andropogon insulare* L., *Digitaria insularis* (L.) Mez ex Ekman, comb. superfl., *Panicum insulare* (L.) G.Mey., Panicum insulare var. typicum Hack., nom. inval., *Panicum lanatum* Rottb., nom. superfl., *Panicum leucophaeum* Kunth, *Milium villosum* Sw., nom. superfl., *Trichachne insularis* (L.) Nees, *Tricholaena insularis* (L.) Griseb., *Syntherisma insularis* (L.) Millsp., *Valota insularis* (L.) Chase.

Herb.

Departmental distribution: Alto Paraguay, Alto Paraná, Amambay, Boquerón, Caaguazú, Central, Cordillera, Guairá, Misiones, Paraguarí, Presidente Hayes, San Pedro.

Voucher: *F. Mereles & S. Soria 9989* (BM, FCQ, G).

***Echinochloa colona*** (L.) Link., Hort. Berol. 2: 209. 1833.

Syn.: Echinochloa colona (L.) Link f. zonalis (Guss.) Wiegand, *Panicum colonum* L., Panicum colonum f. maculatum Arechav., *Oplismenus colonus* (L.) Kunth, *Oplismenus muticus* Phil.

Herb.

Departmental distribution: Alto Paraguay, Amambay, Central, Concepción, Cordillera, Guairá, Itapúa, Misiones, Presidente Hayes.

Voucher: *F. Mereles et al. 10165* (BM, FCQ, G).

***Echinochloa crus-galli*** (L.) P.Beauv. var. ***crus***, Ess. Agrostogr. 161 (53, 169). 1812.

Syn.: *Panicum crus-galli* L.

Herb. Introduced.

Departmental distribution: Alto Paraguay, Central, Cordillera, Presidente Hayes.

Voucher: *F. Mereles & L. Ramella 2925* (FCQ).

***Echinochloa crus-pavonis*** (Kunth) Schult., Mant. 2: 269. 1824.

Syn.: Echinochloa crus-galli (L.) P.Beauv. var. crus-pavonis (Kunth) Hitchc., *Oplismenus crus-pavonis* Kunth, Panicum crus-galli L. var. sabulicola (Nees) Trin.

Herb. Introduced.

Departmental distribution: Alto Paraguay, Alto Paraná, Boquerón, Central, Concepción, Cordillera, Guairá, Itapúa, Misiones, Presidente Hayes.

Voucher: *F. Mereles & J. De Egea 10135* (BM, FCQ, G).

***Echinochloa oryzoides*** (Ard.) Fritsch, Verh. K.K. Zool.-Bot. Ges. Wien 41: 742. 1891.

Syn.: Panicum crus-galli L. var. grandiflorum Döll, *Panicum oryzoides* Ard.

Herb. Introduced.

Departmental distribution: Cordillera.

Voucher: *E. Lurvey 60* (Zuloaga et al. 1994).

***Echinochloa polystachya*** (Kunth) Hitchc. var. ***spectabilis*** (Nees) Mart.Crov., Revista Argent. Agron. 9: 318. 1942.

Syn.: *Echinochloa spectabilis* (Nees ex Trin.) Link, *Panicum spectabile* Nees ex Trin., *Pseudoechinolaena spectabilis* (Nees ex Trin.) Herter

Herb.

Departmental distribution: Alto Paraguay, Central, Cordillera, Guairá, Ñeembucú, Presidente Hayes.

Voucher: *J. De Egea et al. 1311* (FCQ).

***Eleusine indica*** (L.) Gaertn., Fruct. Sem. Pl. 1: 8. 1788.

Syn.: *Cynosurus indicus* L.

Herb. Introduced.

Departmental distribution: Amambay, Caazapá, Central, Guairá, Itapúa, Misiones.

Voucher: *F. Mereles et al. 10162* (FCQ, G).

***Eleusine tristachya*** (Lam.) Lam., Tabl. Encycl. 1(2): 203. 1792.

Syn.: *Cynosurus tristachyos* Lam., Eleusine indica (L.) Gaertn. var. condensata Döll, *Eleusine oligostachya* Link, *Eleusine tristachya* (Lam.) Kunth, comb. superfl.

Herb.

Departmental distribution: Alto Paraná, Central, Guairá, Itapúa, Misiones, Ñeembucú, Misiones, Paraguarí, Presidente Hayes, San Pedro.

Voucher: *J. De Egea et al. 1292* (FCQ).

***Eragrostis airoides*** Nees, Fl. Bras. Enum. Pl. 2(1): 509. 1829.

Syn.: *Agrosticula brasiliensis* (Raddi) Herter, *Aira brasiliensis* Raddi, *Airopsis millegrana* Griseb., *Eragrostis triflora* Ekman, *Poa airoides* (Nees) Kunth, hom. illeg., *Sporobolus brasiliensis* (Raddi) Hack.

Herb.

Departmental distribution: Alto Paraná, Amambay, Central, Guairá, Itapúa, Misiones, Paraguarí, Presidente Hayes, San Pedro.

Voucher: *G. Céspedes et al. 440* (FCQ).

***Eragrostis bahiensis*** Schrad., Mant. 2: 318. 1824.

Syn.: *Eragrostis atrovirens* auct. non (Desf.) Trin., Eragrostis bahiensis Schrad. ex Schult. var. contracta Döll, Eragrostis bahiensis var. laxiuscula Döll, Eragrostis bahiensis f. riparia Burkart, *Eragrostis blepharophylla* Jedwabn., *Eragrostis expansa* Link, *Eragrostis firma* Trin., *Eragrostis macra* Jedwabn., *Eragrostis microstachya* (Link) Link, Eragrostis pilosa (L.) P.Beauv. var. bahiensis (Schrad. ex Schult.) Kuntze, *Eragrostis psammodes* Trin., Eragrostis psammodes var. microstachya (Link) Döll, *Eragrostis squamata* auct. non (Lam.) Steud., *Poa expansa* (Link) Kunth, *Poa microstachya* Link, *Poa psammodes* (Trin.) Kunth.

Herb.

Departmental distribution: Alto Paraná, Amambay, Caaguazú, Central, Concepción, Cordillera, Guairá, Misiones, Paraguarí, Presidente Hayes, San Pedro.

Voucher: *G. Céspedes et al. 448* (FCQ).

***Eragrostis lugens*** Nees, Fl. Bras. Enum. Pl. 2(1): 507. 1829.

Syn.: *Eragrostis flaccida* Lindm., Eragrostis lugens Nees ssp. flaccida (Lindm.) Hack., Eragrostis lugens var. glabrata Döll, Eragrostis lugens f. pallida Hack., Eragrostis pilosa (L.) P.Beauv. var. lugens (Nees) Griseb., *Poa lugens* (Nees) Kunth

Herb.

Departmental distribution: Alto Paraguay, Boquerón, Central, Concepción, Cordillera, Guairá, Ñeembucú, Paraguarí, Presidente Hayes.

Voucher: *G. Céspedes 437* (FCQ).

***Eragrostis pilosa*** (L.) P.Beauv., Ess. Agrostogr. 71 (162, 175). 1812.

Syn.: *Eragrostis damiensiana* (Bonnet) Thell., Eragrostis damiensiana var. condensata (Hack.) Thell., *Eragrostis gracilis* Schrad., *Eragrostis multicaulis* Steud., *Eragrostis peregrina* Wiegand, Eragrostis pilosa (L.) P.Beauv. var. condensata Hack., Eragrostis pilosa ssp. damiensiana (Bonnet) Thell., Eragrostis pilosa var. damiensiana Bonnet, *Eragrostis verticillata* (Cav.) P.Beauv., *Poa pilosa* L.

Herb. Introduced.

Departmental distribution: Alto Paraná, Central, Cordillera.

Voucher: *M. Ortíz 2* (FCQ).

***Hymenachne amplexicaulis*** (Rudge) Nees, Fl. Bras. Enum. Pl. 2(1): 276. 1829.

Syn.: *Agrostis monostachya* Poir., *Panicum amplexicaule* Rudge

Herb.

Departmental distribution: Alto Paraguay, Alto Paraná, Amambay, Boquerón, Caaguazú, Caazapá, Central, Concepción, Cordillera, Guairá, Itapúa, Misiones, Ñeembucú, Paraguarí, Presidente Hayes, San Pedro.

Voucher: *F. Mereles et al. 10180* (FCQ).

***Leptochloa virgata*** (L.) P.Beauv., Ess. Agrostogr. 71 (161, 166). 1812.

Syn.: *Cynodon domingense* (Jacq.) Raspail, *Cynodon virgatus* (L.) Raspail, *Cynosurus dominguensis* Jacq., *Cynosurus virgatus* L., *Eleusine barbata* Desv., *Eleusine domingensis* (Jacq.) Pers., *Eleusine unioloides* Willd. ex Steud., nom. inval., *Eleusine virgata* (L.) Pers., *Diplachne domingensis* (Jacq.) Chapm., *Festuca domingensis* (Jacq.) Lam., *Festuca virgata* (L.) Lam., *Leptochloa barbata* (Desv.) Parodi ex Nicora, *Leptochloa domingensis* (Jacq.) Trin., *Leptochloa mutica* Steud., *Leptochloa procera* Nees, Leptochloa virgata (Jacq.) Chapm. var. domingensis (Jacq.) Link ex Griseb., Leptochloa virgata (L.) P.Beauv. var. mutica (Steud.) Döll, Leptochloa virgata (L.) P.Beauv. var. puberula Hack, *Leptostachys domingensis* (Jacq.) G.Mey.*Rabdochloa domingensis* (Jacq.) P.Beauv.

Herb.

Departmental distribution: Alto Paraguay, Alto Paraná, Amambay, Caaguazú, Caazapá, Central, Concepción, Cordillera, Guairá, Itapúa, Misiones, Paraguarí, Presidente Hayes, San Pedro.

Voucher: *D.R. Brunner 1219* (MO, PY).

***Megathyrsus maximus*** (Jacq.) B.K.Simon & S.W.L.Jacob var. ***maximus***, Austrobaileya 6(3): 572. 2003.

Syn.: *Panicum maximum* Jacq., *Urochloa maxima* (Jacq.) R.D.Webster

Herb. Introduced.

Departmental distribution: Alto Paraguay, Alto Paraná, Amambay, Boquerón, Caazapá, Central, Concepción, Cordillera, Guairá, Itapúa, Paraguarí, Presidente Hayes, San Pedro.

Voucher: *R. Degen 1518* (FCQ).

***Melinis repens*** (Willd.) Zizka, Biblioth. Bot. 138: 55. 1988.

Syn.: *Rhynchelytrum repens* (Willd.) C.E.Hubb., *Rhynchelytrum roseum* (Nees) Stapf & C.E.Hubb., *Saccharum repens* Willd., *Tricholaena repens* (Willd.) Hitchc.

Herb. Introduced.

Departmental distribution: Alto Paraguay, Alto Paraná, Amambay, Canindeyú, Central, Concepción, Cordillera, Guairá, Itapúa, Misiones, Ñeembucú, Paraguarí, Presidente Hayes, San Pedro.

Voucher: *J. De Egea et al. 1271* (FCQ).

***Panicum bergii*** Arechav. var. ***bergii***, Anales Mus. Nac. Montevideo 1: 147. 1894.

Syn.: Panicum bergii Arechav. f. convoluta R.A.Palacios, *Panicum burkartii* Zuloaga, *Panicum pilcomayense* Hack.

Herb.

Departmental distribution: Alto Paraguay, Alto Paraná, Boquerón, Central, Concepción, Misiones, Ñeembucú, Paraguarí, Presidente Hayes.

Voucher: *F. Mereles et al. 10085* (FCQ, G).

***Paspalum acuminatum*** Raddi, Agrostogr. Bras. 25. 1823.

Syn.: *Paspalum serratum* Hitchc. & Chase

Herb.

Departmental distribution: Amambay, Caazapá, Central, Concepción, Cordillera, Itapúa.

Voucher: *A.Schinini 6282* (CTES,G).

***Paspalum almum*** Chase, J. Wash. Acad. Sci. 23(3): 137, f. 1. 1933.

Syn.: *Paspalum hexastachyum* Parodi, Paspalum ovale Nees ex Steud. var. apiculatum Hack.

Herb.

Departmental distribution: Alto Paraguay, Alto Paraná, Central, Cordillera, Misiones, Ñeembucú, Paraguarí, Presidente Hayes.

Voucher: *M. Peña-Chocarro et al. 1669* (FCQ).

***Paspalum chaseanum*** Parodi, J. Wash. Acad. Sci. 23: 137. 1933.

Herb.

Departmental distribution: Alto Paraguay, Boquerón.

Voucher: *F. Mereles & R. Degen 5885* (FCQ).

***Paspalum conjugatum*** P.J.Bergius, Acta Helv. Phys.-Math. 7: 129 (130; t. 8). 1772.

Syn.: *Paspalum renggeri* Steud.

Herb.

Departmental distribution: Alto Paraná, Amambay, Caaguazú, Caazapá, Canindeyú, Central, Concepción, Cordillera, Guairá, Misiones, Paraguarí, San Pedro.

Voucher: *O. Morrone & J. Pensiero 133* (FCQ).

***Paspalum plicatulum*** Michx., Fl. Bor.-Amer. 1: 45. 1803.

Syn.: *Panicum plicatulum* (Michx.) Kuntze, Paspalum lenticulare Kunth f. intumescens (Döll) Killeen, *Paspalum montevidense* Spreng., Paspalum plicatulum Michx. var. genuinum Parodi, nom. inval., Paspalum plicatulum var. glabrum Arechav., Paspalum plicatulum var. intumescens Döll, Paspalum plicatulum var. longipilum Hack., Paspalum plicatulum ssp. montevidense (Spreng.) Roseng., B.R.Arrill. & Izag., Paspalum plicatulum var. villosissimum Pilg., *Paspalum ramosum* Swallen

Herb.

Departmental distribution: Alto Paraguay, Alto Paraná, Amambay, Caaguazú, Central, Concepción, Cordillera, Guairá, Itapúa, Misiones, Ñeembucú, Paraguarí, Presidente Hayes, San Pedro.

Voucher: *J. De Egea et al. 1308* (FCQ).

***Paspalum umbrosum*** Trin., Mém. Acad. Imp. Sci. St.-Pétersbourg, Sér. 6, Sci. Math. 1: 153. 1834.

Syn.: *Paspalum densiflorum* Döll, *Paspalum maritimum* auct. non Trin., Paspalum paniculatum L. ssp. umbrosum (Trin.) Roseng., B.R.Arrill. & Izag.

Herb.

Departmental distribution: Alto Paraná, Caaguazú, Caazapá, Central, Cordillera, Guairá, Itapúa, Misiones, Paraguarí, San Pedro.

Voucher: *F. Mereles et al. 10093* (FCQ).

***Paspalum urvillei*** Steud., Syn. Pl. Glumac. 1(1): 24. 1853.

Syn.: *Paspalum larrañagai* Arechav.

Herb.

Departmental distribution: Alto Paraná, Amambay, Caaguazú, Caazapá, Canindeyú, Central, Concepción, Cordillera, Guairá, Itapúa, Misiones, Ñeembucú, Paraguarí, San Pedro.

Voucher: *E. Lurvey 72* (Zuloaga et al. 2014).

***Paspalum wrightii*** Hitchc. & Chase, Contr. U.S. Natl. Herb. 18: 310. 1917.

Syn.: *Paspalum hydrophilum* Henrard, Paspalum plicatulum Michx. var. multinode Hack, Paspalum virgatum L. var. subplicatum Hack

Herb.

Departmental distribution: Alto Paraguay, Central, Ñeembucú, Presidente Hayes.

Voucher: *J.R. Ramírez 132* (CTES).

***Setaria parviflora*** (Poir.) Kerguélen var. ***parviflora***, Lejeunia 120: 161. 1987.

Syn.: For full list of synonyms see Pensiero (2003).

Herb.

Departmental distribution: Alto Paraguay, Alto Paraná, Amambay, Caaguazú, Canindeyú, Central, Concepción, Cordillera, Guairá, Itapúa, Paraguarí, Presidente Hayes, San Pedro.

Voucher: *F. Mereles et al. 10100* (FCQ).

***Setaria scandens*** Schrad., Mant. 2: 279. 1824.

Syn.: *Chaetochloa scandens* (Schrad.) Scribn., *Panicum scandens* (Schrad.) Trin., Panicum scandens var. grandiflorum Döll, Panicum scandens var. longiseta Döll, Panicum scandens var. vulgare Döll, Setaria scandens Schrad. ex Schult. var. sphacelata Hack., *Setaria trinii* Kunth.

Herb.

Departmental distribution: Amambay, Guairá.

Voucher: *E. Hassler & T. Rojas 10141* (Zuloaga et al. 2014).

***Setaria tenax*** (Rich.) Desv., Opusc. Sci. Phys. Nat. 78. 1831.

Syn.: *Panicum tenax* A.Rich., *Setaria sphaerocarpa* (Salzm.) C.E.Hubb.

Herb.

Departmental distribution: Central, Concepción, Cordillera, Paraguarí.

Voucher: *E.M. Zardini & C. Velázquez 26273* (FCQ, MO).

***Sorghum halepense*** (L.) Pers. var. ***halepense***, Syn. Pl. 1: 101. 1805.

Syn.: *Andropogon halepensis* (L.) Brot., Andropogon sorghum (L.) Brot. var. halepensis (L.) Hack., *Holcus halepensis* L., Sorghum halepense (L.) Pers. var. genuinum Hack., nom. inval.

Herb. Introduced.

Departmental distribution: Alto Paraná, Boquerón, Central, Itapúa.

Voucher: *F. Mereles et al. 10084* (FCQ).

***Sporobolus pyramidatus*** (Lam.) Hitchc., Man. Grasses W. Ind. 84. 1936.

Syn.: *Agrostis pyramidata* Lam., *Sporobolus argutus* (Nees) Kunth, Sporobolus argutus f. purpurascens Hack., Sporobolus argutus var. tuberculatus (Hack.) Hack., *Sporobolus tuberculatus* Hack., *Vilfa arguta* Nees

Herb.

Departmental distribution: Alto Paraguay, Boquerón, Paraguarí, Presidente Hayes.

Voucher: *F. Mereles & S. Soria 9970* (BM, CTES, FCQ, G).

***Stapfochloa elata*** (Desv.) P.M.Peterson, Taxon 64: 460. 2015.

Syn.: *Andropogon barbatus* L., *A polydactylon* L., nom. illeg., *Chloris barbata* (L.) Nash, hom. illeg., *Chloris dandyana* C.D.Adams, *Chloris elata* Desv.,*Chloris polydactyla* (L.) Sw., Chloris polydactyla f. pauciradiata Kurtz

Herb.

Departmental distribution: Alto Paraguay, Alto Paraná, Amambay, Boquerón, Central, Concepción, Cordillera, Guairá, Itapúa, Misiones, Paraguarí, Presidente Hayes.

Voucher: *F. Mereles & S. Soria 9973* (BM, FCQ, G).

*Tragus australianus S.T.Blake, Univ. Queensl. Papers, Dept. Biol. 1 (18): 12. 1941.

Herb. Introduced.

Departmental distribution: Boquerón.

Voucher: *F. Mereles & S. Soria 9942* (FCQ, G).

***Trichloris crinita*** (Lag.) Parodi, Revista Argent. Agron. 14: 63. 1947.

Syn.: *Chloris crinita* Lag., *Chloris mendocina* Phil., *Chloropsis blanchardiana* (E.Fourn. ex Scribn.) Kuntze, *Chloropsis crinita* (Lag.) Kuntze, *Chloropsis mendocina* (Phil.) Kuntze, *Trichloris blanchardiana* E.Fourn. ex Scribn., Trichloris crinita (Lag.) Parodi var. triflora Parodi, Trichloris crinita var. typica Parodi, nom. inval., *Trichloris mendocina* (Phil.) Kurtz, Trichloris mendocina f. blanchardiana (E.Fourn. ex Scribn.) Kurtz, *Trichloris mendocina* (Phil.) Kurtz ex Seckt, hom. illeg.

Herb.

Departmental distribution: Alto Paraguay, Boquerón.

Voucher: *F. Mereles & S. Soria 9976* (FCQ).

***Urochloa brizantha*** (Hochst. ex A.Rich.) R.D.Webster, Austral. Paniceae (Poaceae) 233. 1987.

Syn.: *Brachiaria brizantha* (Hochst. ex A.Rich.) Stapf, *Panicum brizanthum* Hochst. ex A.Rich.

Herb. Introduced.

Departmental distribution: Alto Paraguay, Alto Paraná, Amambay, Canindeyú, Central, San Pedro.

Voucher: *F. Mereles et al. 10075* (FCQ).

***Urochloa decumbens*** (Stapf) R.D.Webster, Austral. Paniceae (Poaceae) 234. 1987.

Herb. Introduced.

Departmental distribution: Cordillera, San Pedro.

Voucher: *O. Morrone & J. Pensiero 371* (FCQ).

***Urochloa paucispicata*** (Morong) Morrone & Zuloaga, Darwiniana 31: 95. 1992.

Syn.: *Acroceras paucispicata* (Morong) Henrard, *Brachiaria paucispicata* (Morong) Clayton, *Panicum paucispicatum* Morong

Herb.

Departmental distribution: Alto Paraguay, Boquerón, Central, Concepción, Cordillera, Presidente Hayes.

Voucher: *F. Mereles & S. Soria 9943* (BM, CTES, FCQ, G).

***Urochloa plantaginea*** (Link.) R.D.Webster, Syst. Bot. 13(4): 607. 1988.

Syn.: *Brachiaria plantaginea* (Link) Hitchc., *Panicum plantagineum* Link

Herb.

Departmental distribution: Alto Paraguay, Central, Concepción, Cordillera, Guairá, San Pedro.

Voucher: *J. De Egea et al. 1291* (FCQ).

#### POLYGONACEAE

***Polygonum acuminatum*** Kunth, Nov. Gen. Sp. 2: 178. 1817.

Syn.: *Persicaria acuminata* (Kunth) M.Gómez, Polygonum acuminatum Kunth var. glabrescens Meisn., Polygonum acuminatum var. microstemom Mart. ex Meisn., Polygonum acuminatum var. setigerum (Wedd.) Meisn., *Polygonum alfredi* Pilg., *Polygonum bettfreundianum* Lindau, *Polygonum floribundum* Wedd., *Polygonum setigerum* Wedd.

Herb.

Departmental distribution: Alto Paraguay, Alto Paraná, Amambay, Boquerón, Caaguazú, Caazapá, Canindeyú, Central, Concepción, Cordillera, Guairá, Itapúa, Misiones, Ñeembucú, Paraguarí, Presidente Hayes, San Pedro.

Voucher: *A. Schinini & R. Palacios 25915* (FCQ).

***Polygonum aviculare*** L., Sp. Pl. 1: 362 (-363). 1753.

Syn.: *Polygonum arenastrum* Boreau, *Polygonum berteroi* Phil., *Polygonum striatum* K.Koch, *Polygonum uruguense* H.Gross

Herb. Introduced.

Departmental distribution: Presidente Hayes.

Voucher: *T. Rojas 409* (Cialdella and Brandbyge 2001).

***Polygonum convolvulus*** L., Sp. Pl. 1: 364. 1753.

Syn.: *Bilderdykia convolvulus* (L.) Dumort., *Fallopia convolvulus* (L.) A.Löve, *Tiniaria convolvulus* (L.) Webb & Moq.

Herb. Introduced.

Departmental distribution: Cordillera, Guairá, Itapúa.

Voucher: *E. Lurvey 17* (CTES).

***Polygonum hispidum*** Kunth, Nov. Gen. Sp. (quarto ed.) 2: 178. 1817.

Syn.: *Persicaria hispida* (Kunth) M.Gómez

Herb.

Departmental distribution: Alto Paraguay, Boquerón, Central, Cordillera, Ñeembucú, Presidente Hayes.

Voucher: *F. Mereles et al. 10117* (FCQ, G).

***Polygonum hydropiperoides*** Michx. var. ***setaceum*** (Baldwin) Gleason, Phytologia 4(1): 23. 1952.

Syn.: Polygonum hydropiperoides Michx. var. virgatum (Cham. & Schltdl.) Meisn., *Polygonum setaceum* Baldwin, *Polygonum virgatum* Cham. & Schltdl.

Herb.

Departmental distribution: Caazapá, Central, Cordillera, Guairá, Itapúa, Misiones, Ñeembucú, Paraguarí, Presidente Hayes, San Pedro.

Voucher: *J. De Egea et al. 691* (BM, FCQ).

***Polygonum lapathifolium*** L., Sp. Pl. 1: 360. 1753.

Syn.: *Persicaria laphatifolia* (L.) Delarbre, Polygonum ferrugineum Wedd. var. patagonicum (Speg.) Macloskie, *Polygonum lanigerum* R.Br., Polygonum persicaria L. var. vernicosum Cham. & Schltdl., Polygonum spectabile Mart. ex Meisn. var. patagonica Speg., *Polygonum utriculatum* J.Remy

Herb. Introduced.

Departmental distribution: Concepción, Presidente Hayes.

Voucher: *F. Mereles 1387* (FCQ).

***Polygonum meisnerianum*** Cham. & Schltdl., Linnaea 3(1): 40–42. 1828.

Syn.: *Persicaria meisneriana* (Cham. & Schltdl.) M.Gómez, *Polygonum beyrichianum* Cham. & Schltdl., *Polygonum chamissoeanum* Wedd., Polygonum meisnerianum Cham. & Schltdl. var. beyrichianum (Cham. & Schltdl.) Meisn., Polygonum meisnerianum var. setosum Chodat & Hassl.

Herb.

Departmental distribution: Alto Paraná, Amambay, Caazapá, Central, Cordillera, Guairá, Itapúa, Misiones, Ñeembucú, Paraguarí, San Pedro.

Voucher: *R. Degen 9* (FCQ).

***Polygonum punctatum*** Elliot, Sketch Bot. S. Carolina 1(5): 455–456. 1821.

Syn.: *Persicaria punctata* (Elliot) Small, *Polygonum acre* Kunth, hom. illeg., *Polygonum epilobioides* Wedd., *Polygonum hydropiperoides* Pursh, hom. illeg.

Herb.

Departmental distribution: Alto Paraguay, Alto Paraná, Amambay, Caaguazú, Caazapá, Canindeyú, Central, Concepción, Cordillera, Guairá, Itapúa, Misiones, Ñeembucú, Paraguarí, Presidente Hayes, San Pedro.

Voucher: *F. Mereles et al. 10019* (BM, CTES, FCQ, G).

***Rumex obovatus*** Danser, Ned. Kruidk. Arch. 1920: 241. 1921.

Syn.: *Rumex paraguayensis* auct. non D.Parodi

Herb.

Departmental distribution: Alto Paraná, Central, Guairá, Presidente Hayes.

Voucher: *F. Mereles et al. 10092* (FCQ, G).

***Rumex paraguayensis*** D.Parodi, Anales Soc. Ci. Argent. 5: 160. 1878.

Herb.

Departmental distribution: Alto Paraguay, Boquerón, Central, Cordillera, Misiones, Presidente Hayes.

Voucher: *R. Spichiger et al. RS2209* (FCQ).

***Rumex pulcher*** L., Sp. Pl. 1: 336. 1753.

Herb. Introduced.

Departmental distribution: Alto Paraná, Central, Cordillera, Misiones.

Voucher: *I. Basualdo 1164* (FCQ).

#### PORTULACACEAE

***Portulaca cryptopetala*** Speg., Anales Soc. Ci. Argent. 82: 217. 1916.

Syn.: Portulaca cryptopetala Speg. var. diversifolia (Poelln.) D.Legrand, Portulaca cryptopetala var. legrandii (Poelln.) D.Legrand, Portulaca cryptopetala f. phenopetala Speg., *Portulaca diversifolia* D.Legrand, nom. illeg., Portulaca diversifolia Poelln. var. legrandii (Poelln.) D.Legrand *Portulaca legrandii* Poelln.

Herb.

Departmental distribution: Alto Paraguay, Central, Ñeembucú, Paraguarí, Presidente Hayes, San Pedro.

Voucher: *F. Mereles 10125* (FCQ).

***Portulaca oleracea*** L., Sp. Pl. 1: 445. 1753.

Syn.: Portulaca cryptopetala Speg. var. poellnitziana (D.Legrand) D.Legrand, *Portulaca marginata* Kunth, *Portulaca neglecta* Mack. & Bush, Portulaca oleracea L. var. granulato-stellulata Poelln., Portulaca oleracea var. macrantha Eggers, Portulaca oleracea var. micrantha Eggers, Portulaca oleracea var. parviflora (Haw.) Griseb., Portulaca oleracea var. silvestris (Montandon) Thell., *Portulaca poellnitziana* D.Legrand, *Portulaca retusa* Engelm.

Herb. Introduced.

Departmental distribution: Alto Paraguay, Boquerón, Central, Paraguarí, Presidente Hayes, San Pedro.

Voucher: *F. Mereles & S. Soria 9994* (FCQ).

#### RUBIACEAE

***Borreria eryngioides*** Cham. & Schltdl. var. ***affinis*** (DC.) K.Schum., Fl. Bras. 6(6): 48. 1888.

Syn.: *Borreria affinis* DC., *Borreria assurgens* (Ruiz & Pav.) Griseb., Borreria assurgens Hassl. var. longisepala Hassl., *Borreria chacoensis* Hassl., Borreria chacoensis var. glabrata Hassl., Borreria eryngioides Cham. & Schltdl. var. latifolia Hassl., Borreria parviflora G.Mey. var. scabra Griseb.

Herb or subshrub.

Departmental distribution: Alto Paraguay, Alto Paraná, Caazapá, Central, Guairá, Presidente Hayes.

Voucher: *F. Mereles & S. Soria 10204* (FCQ, G).

***Borreria verticillata*** (L.) G.Mey., Prim. Fl. Esseq. 83. 1818.

Syn.: Borreria capitata (Ruiz & Pav.) DC. f. ferruginea auct. non (A.St.-Hil.) Steyerm., *Spermacoce verticillata* L.

Herb or subshrub.

Departmental distribution: Alto Paraná, Amambay, Caaguazú, Caazapá, Canindeyú, Central, Concepción, Cordillera, Guairá, Misiones, Ñeembucú, Paraguarí, Presidente Hayes, San Pedro.

Voucher: *J. De Egea et al. 1242* (BM, FCQ).

***Mitracarpus megapotamicus*** (Spreng.) Kuntze, Publ. Field Mus. Nat. Hist., Bot. Ser. 7: 331. 1931.

Syn.: *Mitracarpium cuspidatum* DC., *Mitracarpus megapotamicus* (Spreng.) Standl., comb. superfl., *Mitracarpium peladilla* Griseb., *Mitracarpus sellowianum* Cham. & Schltdl., *Spermacoce megapotamica* Spreng.

Herb.

Departmental distribution: Alto Paraguay, Amambay, Boquerón, Caaguazú, Caazapá, Central, Concepción, Guairá, Misiones, Paraguarí, Presidente Hayes.

Voucher: *F. Mereles et al. 10021* (FCQ).

***Mitracarpus villosus*** (Sw.) DC., Prodr. 4: 572. 1830.

Syn.: *Mitracarpus hirtus* auct. non (L.) DC., *Spermacoce hirta* auct. non L., *Spermacoce villosa* Sw.

Herb.

Departmental distribution: Caaguazú, Caazapá, Canindeyú, Central, Paraguarí.

Voucher: *F. Mereles et al. 10103* (FCQ).

*Oldenlandia lancifolia (Schumach.) DC., Prodr. 4: 425. 1830.

Syn.: *Hedyotis lancifolia* Schumach.

Herb. Introduced.

Departmental distribution: Central, Cordillera.

Voucher: *F. Mereles 10120* (FCQ).

***Richardia brasiliensis*** Gomes, Mem. sobre Ipecac. 31. 1801.

Syn.: *Richardsonia brasiliensis* (Gomes) Hayne, *Richardia pilosa* auct. non Ruiz & Pav., *Richardsonia rosea* A.St.-Hil., *Richardia rosea* (A.St.-Hil.) Schult.f., *Richardia scabra* auct. non L., Richardsonia brasiliensis (Gomes) Hayne var. dubia Beauverd & Felipp., *Richardsonia scabra* auct. non (L.) A.St.-Hil.

Herb.

Departmental distribution: Alto Paraguay, Alto Paraná, Amambay, Boquerón, Caaguazú, Caazapá, Canindeyú, Central, Concepción, Cordillera, Guairá, Itapúa, Ñeembucú, Paraguarí, Presidente Hayes, San Pedro.

Voucher: *J. De Egea et al. 1234* (FCQ).

***Staelia virgata*** (Link ex Roem. & Schult.) K.Schum. var. ***virgata***, Fl. Bras. 6(6): 76. 1888.

Syn.: *Borreria finitima* S.Moore, *Mitracarpus virgatus* (Link ex Roem. & Schult.) Cham. & Schltdl., *Spermacoce virgata* Link ex Roem. & Schult., *Staelia caespitosa* Griseb.

Herb.

Departmental distribution: Alto Paraguay, Concepción, Cordillera, Misiones, Ñeembucú, Paraguarí, Presidente Hayes.

Voucher: *F. Mereles & J. De Egea 10152* (FCQ, G).

#### SOLANACEAE

***Nicotiana glauca*** Graham, Edinburgh New Philos. J. 5: 175. 1828.

Syn.: *Acnistus virgatus* Griseb., Nicotiana glauca Graham var. angustifolia Comes, Nicotiana glauca var. decurrens Comes, Nicotiana glauca var. grandiflora Comes, Nicotiana glauca f. lateritia Lillo, Nicotiana glauca var. typica Millán, nom. inval., *Nicotidendron glauca* (Graham) Griseb., *Siphaulax glabra* Raf.

Shrub or subshrub.

Departmental distribution: Alto Paraguay, Boquerón, Central, Cordillera, Ñeembucú, Paraguarí.

Voucher: *F. Mereles et al. 10156* (BM, FCQ, G).

***Nicotiana longiflora*** Cav., Descr. Pl. 106. 1802.

Syn.: *Nicotiana acuta* Griseb., *Nicotiana acutiflora* A.St.-Hil., Nicotiana longiflora Cav. var. acutiflora (A.St.-Hil.) Comes, Nicotiana longiflora var. breviflora Comes, nom. inval., Nicotiana longiflora var. grandifolia Morong

Herb.

Departmental distribution: Boquerón, Central, Concepción, Cordillera, Paraguarí, Presidente Hayes, San Pedro.

Voucher: *F. Mereles et al. 10055* (FCQ).

***Physalis angulata*** L., Sp. Pl. 1: 183. 1753.

Herb.

Departmental distribution: Alto Paraguay, Amambay, Boquerón, Guairá, Presidente Hayes, San Pedro.

Voucher: *J. De Egea et al. 1319* (BM, FCQ).

***Solanum americanum*** Mill., Gard. Dict. (ed. 8) no. 5. 1768.

Syn.: *Solanum adventitium* Polg., Solanum americanum Mill. var. nodiflorum (Jacq.) Edmonds, *Solanum sciaphilum* Bitter, *Solanum curtipes* Bitter, Solanum nigrum L. var. americanum (Mill.) O.E.Schulz, Solanum nigrum var. virginicum L., *Solanum nodiflorum* Jacq., Solanum nodiflorum var. acuminatum Chodat, Solanum nodiflorum var. micropyllum Hassl., Solanum nodiflorum var. petiolastrum Dunal, Solanum nodiflorum var. sapucayense Chodat, *Solanum oleraceum* Dunal, *Solanum tenellum* Bitter.

Herb or subshrub.

Departmental distribution: Alto Paraguay, Alto Paraná, Amambay, Boquerón, Caaguazú, Caazapá, Canindeyú, Central, Concepción, Cordillera, Guairá, Itapúa, Misiones, Ñeembucú, Paraguarí, Presidente Hayes, San Pedro.

Voucher: *J. De Egea et al. 1320* (BM, FCQ).

***Solanum chacoense*** Bitter, Repert. Spec. Nov. Regni Veg. 11: 18. 1912.

Syn.: *Solanum arnezii* Cárdenas, *Solanum bitteri* Hassl., *Solanum boergeri* Bukasov, *Solanum caipipendense* Cárdenas, *Solanum calvescens* Bitter, Solanum chacoense var. angustisectum (Hassl.) Hassl., Solanum chacoense f. caipipendense (Cárdenas) Correll, Solanum chacoense f. gibberulosum (Juz. & Bukasov) Correll, Solanum chacoense f. glabrescens (Hassl.) Hassl., Solanum chacoense var. latisectum (Hassl.) Hassl., Solanum chacoense subsp. muelleri (Bitter) Hawkes, Solanum chacoense f. plurijugum Hassl., Solanum chacoense f. puberulum Hassl., Solanum chacoense subsp. subtilius (Bitter) Hawkes, Solanum commersonii var. glabriusculum J.Rémy, *Solanum cuevoanum* Cárdenas, *Solanum dolichostigma* Buk. ex Lechn., *Solanum emmeae* Juz. & Bukasov, *Solanum garciae* Juz. & Bukasov, *Solanum gibberulosum* Juz. & Bukasov, *Solanum guaraniticum* Hassl., Solanum guaraniticum var. angustisectum Hassl., Solanum guaraniticum f. glabrescens Hassl., Solanum guaraniticum var. latisectum Hassl., *Solanum horovitzii* Bukasov, Solanum horovitzii var. glabristylum Hawkes, Solanum horovitzii var. multijugum Hawkes, Solanum jamesii var. grandifrons Bitter, *Solanum jujuyense* Hawkes, *Solanum knappei* Juz. & Bukasov, *Solanum laplaticum* Bukasov, *Solanum limense* Correll, *Solanum muelleri* Bitter, Solanum muelleri f. densipilosum Correll, *Solanum parodii* Juz. & Bukasov, *Solanum pseudomaglia* L.Planch., *Solanum saltense* Hawkes, *Solanum schickii* Juz. & Bukasov, *Solanum subtilius* Bitter, Solanum tuberosum subsp. yanacochense Ochoa, *Solanum yanacochense* (Ochoa) Gorbat., *Solanum yungasense* Hawkes.

Herb.

Departmental distribution: Boquerón, Central, Concepción, Cordillera, Guairá, Itapúa, Paraguarí, Presidente Hayes, San Pedro.

Voucher: *I. Basualdo 270* (FCQ).

***Solanum granuloso-leprosum*** Dunal, Prodr. 13(1): 115. 1852.

Syn.: *Solanum auriculatum* auct. non Aiton, Solanum verbascifolium f. eupulverulentum Hassl., Solanum verbascifolium L. f. granuloso-leprosum (Dunal) Hassl.

Shrub or tree.

Departmental distribution: Alto Paraguay, Alto Paraná, Amambay, Caaguazú, Caazapá, Canindeyú, Central, Concepción, Cordillera, Guairá, Itapúa, Misiones, Ñeembucú, Paraguarí, Presidente Hayes, San Pedro.

Voucher: *J. De Egea et al. 1310* (FCQ).

***Solanum guaraniticum*** A.St.-Hil., Mém. Mus. Hist. Nat. 12: 321. 1825.

Syn.: Solanum bonariense L. f. intermedium Hassl., Solanum bonariense f. leptophyllum Chodat, Solanum bonariense f. lobatum (Chodat) Hassl., Solanum bonariense var. paraguariense (Chodat) Chodat, Solanum bonariense f. subintegrum (Hassl.) Chodat, nom. illeg., Solanum bonariense f. typicum Hassl., nom. inval., Solanum bonariense var. villaricense (Morong) Chodat, Solanum fastigiatum Willd. var. acicularium Dunal, *Solanum paraguariense* Chodat, *Solanum villaricense* Morong, Solanum villaricense f. lobatum Hassl., Solanum villaricense var. paraguariense (Chodat) Hassl., Solanum villaricense f. subintegrum Hassl., Solanum villaricense Hassl., nom. inval. var. typicum.

Shrub.

Departmental distribution: Alto Paraná, Caaguazú, Caazapá, Canindeyú, Central, Guairá, Itapúa, Misiones, Paraguarí, Presidente Hayes.

Voucher: *F. Mereles et al. 10024* (BM, FCQ, G).

***Solanum palinacanthum*** Dunal, Prodr. 13(1): 245. 1852.

Syn.: *Solanum claviceps* Griseb., Solanum palinacanthum Dunal var. acutilobum Dunal, Solanum palinacanthum f. velutinum Chodat, *Solanum vexans* S.Moore

Shrub or small tree.

Departmental distribution: Alto Paraguay, Alto Paraná, Amambay, Caaguazú, Caazapá, Canindeyú, Central, Concepción, Cordillera, Guairá, Itapúa, Misiones, Ñeembucú, Paraguarí, Presidente Hayes, San Pedro.

Voucher: *F. Mereles et al. 10014* (BM, FCQ, G).

***Solanum pseudocapsicum*** L., Sp. Pl. 1: 184. 1753.

Syn.: *Solanum capsicastrum* Link ex Schauer, Solanum capsicastrum var. caaguazuense Chodat, *Solanum diflorum* Vell., Solanum diflorum var. angustifolium Kuntze, Solanum ipecacuanha Chodat var. calvescens Chodat, *Solanum pavimenti* L.B.Sm. & Downs, Solanum pseudocapsicum var. ambiguum Hassl., Solanum pseudocapsicum L. f. calvescens (Chodat) Hassl., Solanum pseudocapsicum ssp. diflorum (Vell.) Hassl., Solanum pseudocapsicum var. hygrophilum (Schltdl.) Hassl., Solanum pseudocapsicum var. normale Kuntze, Solanum pseudocapsicum var. parvifolium Kuntze, Solanum pseudocapsicum f. pilosum Kuntze, Solanum pseudocapsicum f. pilosulum Hassl., Solanum pseudocapsicum var. sendtnerianum Hassl., Solanum pseudocapsicum var. typicum Hassl., nom. inval.

Subshrub.

Departmental distribution: Alto Paraguay, Amambay, Caaguazú, Caazapá, Canindeyú, Central, Cordillera, Guairá, Misiones, Ñeembucú, Paraguarí, Presidente Hayes, San Pedro.

Voucher: *J. De Egea et al. 1263* (FCQ).

***Solanum sisymbriifolium*** Lam., Tabl. Encycl. 2: 25. 1794.

Syn.: *Solanum balbisii* Dunal, Solanum balbisii var. bipinnata Hook., Solanum balbisii var. oligospermum Sendtn., Solanum balbisii var. purpureum Hook., *Solanum bipinnatifidum* Larrañaga, *Solanum decurrens* Balb., Solanum sisymbriifolium f. albiflorum Kuntze, Solanum sisymbriifolium Lam. var. brevilobum Dunal, Solanum sisymbriifolium var. heracleifolium Sendtn., nom. inval., Solanum sisymbriifolium f. lilacinum Kuntze, Solanum sisymbriifolium var. macrocarpum Kuntze, Solanum sisymbriifolium var. oligospermum (Sendtn.) Dunal, Solanum sisymbriifolium var. purpureiflorum Dunal, *Solanum subviscidum* Schrank

Herb or subshrub.

Departmental distribution: Alto Paraguay, Boquerón, Caaguazú, Central, Cordillera, Guairá, Itapúa, Ñeembucú, Paraguarí, Presidente Hayes, San Pedro.

Voucher: *F. Mereles et al. 10015* (FCQ, G).

***Solanum tweedieanum*** Hook., Bot. Mag. 62: t.3385. 1835.

Syn.: *Solanum atriplicoides* Herter, *Solanum meizonanthum* Bitter, *Solanum physalidicalyx* Bitter, Solanum physalidicalyx var. integrescens Bitter, Solanum physalidicalyx var. plurilobulatum Bitter, *Solanum sidifolium* Speg.

Herb.

Departmental distribution: Alto Paraguay, Boquerón, Presidente Hayes.

Voucher: *F. Mereles & S. Soria 9992* (FCQ, G, BM, CTES).

***Solanum viarum*** Dunal, Prodr. 13(1): 240. 1852.

Syn.: *Solanum chloranthum* DC., hom. illeg., *Solanum reflexum* auct. non Schrank, *Solanum viridiflorum* Schltdl.

Shrub.

Departmental distribution: Alto Paraguay, Alto Paraná, Boquerón, Caazapá, Canindeyú, Central, Concepción, Cordillera, Guairá, Ñeembucú, Paraguarí, San Pedro.

Voucher: *F. Mereles & S. Soria 9974* (FCQ).

#### TALINACEAE

***Talinum fruticosum*** (L.) Juss., Gen. Pl. 312. 1789.

Syn.: *Portulaca fruticosa* L., *Portulaca racemosa* L., *Portulaca triangularis* Jacq., *Talinum racemosum* (L.) Rohrb., *Talinum triangulare* (Jacq.) Willd.

Subshrub. Introduced.

Departmental distribution: Alto Paraguay, Boquerón, Cordillera, Paraguarí, Presidente Hayes, San Pedro.

Voucher: *F. Mereles & S. Soria 9963* (BM, CTES, FCQ, G).

***Talinum paniculatum*** (Jacq.) Gaertn., Fruct. Sem. Pl. 2: 219. 1791.

Syn.: *Portulaca paniculata* Jacq., *Portulaca patens* L., *Talinum patens* (L.) Willd.

Herb. Introduced.

Departmental distribution: Alto Paraguay, Amambay, Boquerón, Caazapá, Canindeyú, Central, Cordillera, Guairá, Paraguarí, Presidente Hayes, San Pedro.

Voucher: *M. Peña-Chocarro & J. De Egea 2453* (BM, CTES, FCQ, MO).

#### URTICACEAE

***Parietaria debilis*** G.Forst., Fl. Ins. Austr. 73. 1786.

Syn.: *Freirea erecta* Phil., *Freirea humifusa* Gay, Parietaria debilis G.Forst. var. diffusa Wedd., Parietaria debilis var. gracilis (Lowe) Wedd., Parietaria debilis var. micrantha (Ledeb.) Wedd., *Parietaria fernandeziana* (Steud.) L.E.Navas, *Parietaria gracilis* Lowe, *Parietaria humifusa* (Gay) Blume, *Parietaria micrantha* Ledeb., *Urtica fernandeziana* Steud., *Urtica parietariaefolia* Bertero ex Steud.

Herb.

Departmental distribution: Alto Paraguay, Alto Paraná, Amambay, Caazapá, Central, Concepción, Cordillera, Guairá, Paraguarí.

Voucher: *J. De Egea 1295* (BM, FCQ, G).

#### VERBENACEAE

***Aloysia virgata*** (Ruiz & Pav.) Pers. var. ***platyphylla*** (Briq.) Moldenke, Phytologia 2: 310. 1947.

Syn.: *Aloysia naviculata* Ravenna, Aloysia virgata (Ruiz & Pav.) Pers. var. elliptica (Briq.) Moldenke, Lippia virgata (Ruiz & Pav.) Steud. var. elliptica Briq., Lippia virgata var. platyphylla Briq.

Shrub.

Departmental distribution: Alto Paraguay, Alto Paraná, Amambay, Central, Concepción, Cordillera, Guairá, Itapúa, Misiones, Ñeembucú, Paraguarí, Presidente Hayes, San Pedro.

Voucher: *F. Mereles 9928* (BM, FCQ, G).

*Glandularia cabrerae (Moldenke) Botta, Bol. Soc. Argent. Bot. 26: 243. 1990.

Syn.: *Verbena cabrerae* Moldenke, Verbena dissecta Willd. ex Spreng. f. alba Moldenke

Herb.

Departmental distribution: Alto Paraguay, Alto Paraná.

Voucher: *F. Mereles et al. 10067* (FCQ).

***Glandularia tomophylla*** (Briq.) P.Peralta, Darwiniana 45: 241. 2007.

Syn.: *Glandularia spectabilis* (Moldenke) Botta, *Verbena calliantha* Briq., Verbena calliantha var. microsoma Briq., Verbena megapotamica Spreng. var. pinnatiloba Kuntze, *Verbena pinnatiloba* (Kuntze) Moldenke, *Verbena ramboi* Moldenke, *Verbena spectabilis* Moldenke, *Verbena storeoclada* Briq., *Verbena tomophylla* Briq.

Herb.

Departmental distribution: Alto Paraguay, Alto Paraná, Caaguazú, Central, Guairá, Itapúa, Misiones, Ñeembucú, Paraguarí.

Voucher: *J. De Egea et al. 1136* (BM, FCQ, G).

***Glandularia tweediana*** (Niven ex Hook.) P.Peralta, Ann. Missouri Bot. Gard. 98(3): 400. 2011.

Syn.: *Glandularia incisa* (Hook.) Tronc., *Verbena incisa* Hook., Verbena megapotamica Spreng. var. truncatula Briq., Verbena megapotamica var. tweediana (Niven ex Hook.) Kuntze, *Verbena tweediana* Niven ex Hook., Verbena tweedieana var. arriana Niven ex Maund.

Herb.

Departmental distribution: Alto Paraguay, Caaguazú, Central, Cordillera, Guairá, Ñeembucú, Paraguarí, Presidente Hayes, San Pedro.

Voucher: *J. De Egea et al. 1274* (BM, FCQ).

***Stachytarpheta cayennensis*** (Rich.) Vahl, Enum. Pl. 1: 208. 1804.

Syn.: *Stachytarpheta australis* Moldenke, Stachytarpheta cayennensis (Rich.) Vahl f. alba Moldenke ex Tronc. & Botta, Stachytarpheta cayennensis f. albiflora Moldenke, Stachytarpheta cayennensis var. candicans Briq., Stachytarpheta cayennensis var. virescens Briq., *Stachytarpheta dichotoma* (Ruiz & Pav.) Vahl, Stachytarpheta maximiliani Schauer var. ciliaris Moldenke, *Stachytarpheta patens* Moldenke, *Verbena cayennensis* Rich., *Verbena dichotoma* Ruiz & Pav.

Herb or subshrub.

Departmental distribution: Alto Paraguay, Alto Paraná, Amambay, Caaguazú, Caazapá, Canindeyú, Central, Concepción, Cordillera, Guairá, Itapúa, Misiones, Ñeembucú, Paraguarí, Presidente Hayes, San Pedro.

Voucher: *J. De Egea et al. 1221* (BM, FCQ).

***Verbena litoralis*** Kunth var. ***litoralis***, Nov. Gen. Sp. 2: 276, t. 137. 1818.

Syn.: *Verbena affinis* M.Martens & Galeotti, *Verbena approximata* Briq., Verbena bonariensis L. var. litoralis (Kunth) Gillies & Hook. ex Hook.,*Verbena caracasana* Kunth, *Verbena cordobensis* Briq., *Verbena integrifolia* Sessé & Moç., Verbena integrifolia f. albiflora Chodat, Verbena litoralis Kunth var. albiflora Moldenke, Verbena litoralis f. albiflora (Moldenke) Moldenke Verbena litoralis f. angustifolia Chodat,Verbena litoralis var. caracasana (Kunth) Briq., Verbena litoralisvar. glabrior Benth., Verbena litoralis var. leptostyachya Schauer, Verbena litoralis var. pycnostachya Schauer, nom. inval., *Verbena nudiflora* Nutt. ex Turcz.

Herb.

Departmental distribution: Alto Paraná, Amambay, Caaguazú, Caazapá, Canindeyú, Central, Concepción, Cordillera, Guairá, Itapúa, Misiones, Ñeembucú, Paraguarí, Presidente Hayes, San Pedro.

Voucher: *F. Mereles et al. 10091* (FCQ, G).

***Verbena montevidensis*** Spreng., Syst. Veg. (ed. 16) 2:747. 1825.

Syn.: *Verbena isabellei* Briq., *Verbena minutiflora* Briq. ex Moldenke, Verbena minutiflora var. peruviana Moldenke.

Herb.

Departmental distribution: Alto Paraguay, Caaguazú, Caazapá, Central, Cordillera, Guairá, Itapúa, Misiones, Paraguarí, Presidente Hayes.

Voucher: *R. Degen et al. 1729* (FCQ).
